# Quantum noise spectroscopy of nanoscale charge defects in silicon carbide at room temperature

**DOI:** 10.1126/sciadv.aej5462

**Published:** 2026-09-25

**Authors:** Jinpeng Liu, Yuanhong Teng, Yu Chen, Yixuan Wang, Sen Luan, Chihang Luo, Jun Yin, Hao Li, Lixing You, Qiang Li, Chunming Yin, Ya Wang, Qi Zhang, Fazhan Shi

**Affiliations:** ^1^ School of Biomedical Engineering, Division of Life Sciences and Medicine, University of Science and Technology of China, Hefei, Anhui 230026, China.; ^2^ Laboratory of Spin Magnetic Resonance, School of Physical Sciences, Anhui Province Key Laboratory of Scientific Instrument Development and Application, University of Science and Technology of China, Hefei 230026, China.; ^3^ Suzhou Institute for Advanced Research, University of Science and Technology of China, Suzhou, Jiangsu 215123, China.; ^4^ Hefei National Laboratory, University of Science and Technology of China, Hefei 230088, China.; ^5^ Shanghai Key Laboratory of Superconductor Integrated Circuit Technology, Shanghai Institute of Microsystem and Information Technology, Chinese Academy of Sciences, Shanghai 200050, China.; ^6^ State Key Laboratory of Silicon Materials and Advanced Semiconductors & School of Materials Science and Engineering, Institute of Advanced Semiconductors, ZJU-Hangzhou Global Scientific and Technological Innovation Center, Zhejiang University, Hangzhou, Zhejiang 310027, China.; ^7^ Hefei National Research Center for Physical Sciences at the Microscale, University of Science and Technology of China, Hefei 230026, China.; ^8^ Institute of Quantum Sensing, School of Physics, Institute of Fundamental and Transdisciplinary Research, Zhejiang Key Laboratory of R&D and Application of Cutting-edge Scientific Instruments, Zhejiang University, Hangzhou 310027, China.

## Abstract

The nanoscale charge environment critically influences the performance of semiconductor devices, yet traditional characterization techniques usually lack the spatial resolution to resolve nanoscale charge heterogeneity and identify microscopic noise sources. Here, we address this critical gap using individual PL5 color centers in commercial silicon carbide as room-temperature, broadband (near-dc to gigahertz) quantum spectrometers. We report real-time, nanoscale observation of single-charge tunneling dynamics at room temperature in 4H-SiC by monitoring the random telegraph noise using optically detected magnetic resonance. This capability enables an electrical noise imaging technique that shows distinct noise variations on different wafers. Using dynamical decoupling and $T_{1}$ relaxation spectroscopy, we observe correlations of noise spectral density across frequency bands and obtain the nanoscale electron paramagnetic resonance fingerprint of spin defects in silicon carbide. Ultimately, through surface chemical engineering, we identify the native oxide layer as the main source of resolved electromagnetic fluctuations. This work establishes a versatile atomic-scale metrology platform bridging quantum sensing and semiconductor engineering, offering critical insights for optimizing third-generation semiconductor interfaces and advancing solid-state quantum technologies.

## INTRODUCTION

Silicon carbide (SiC) is recognized as a promising semiconductor material due to its outstanding physical properties, such as high thermal conductivity and wide bandgap, making it ideal for the fabrication of high-power devices (*1*). However, the performance and reliability of SiC-based complementary metal-oxide semiconductor (CMOS) devices are fundamentally limited by atomic-scale defects in bulk and at the interface, which act as noise sources, reduce carrier lifetimes, and trigger breakdown (*2*). Despite the maturity of SiC fabrication, characterizing the microscopic electric and magnetic environments associated with these defects presents a critical challenge. Conventional methods such as standard deep-level transient spectroscopy (DLTS), electron spin resonance, and macroscopic photoluminescence (PL) typically provide volume-averaged metrics (*3*), inherently preventing the identification and localization of noise sources at the nanoscale.

To fill this gap, solid-state quantum sensors have emerged as promising tools for nanoscale diagnostics. While nitrogen-vacancy (NV) centers in diamond are widely used for nanoscale electric field sensing (*4*–*13*), their application to SiC is constrained by the sensor-sample distance and the inability to probe the intrinsic bulk and interface environment of the semiconductor (*14*–*16*). Moreover, single-charge tunneling events detected via PL excitation predominantly required cryogenic temperatures, thereby limiting their applicability to in-fab-line monitoring (*10*, *17*–*19*). To achieve true in situ monitoring, we focus on using intrinsic point defects within the 4H-SiC lattice itself. Among these, the PL5 center stands out not only for its optical addressability and spin coherence (*20*–*23*) but also for its giant Stark effect. The Stark coupling parameters of PL5 center are two to seven times larger than those of NV centers in diamond (*24*), making it an ultrasensitive electrometer capable of detecting local electric field fluctuations. This distinct property makes PL5 center an ideal candidate for imaging the noise landscape inside SiC devices at room temperature.

Concurrently, recent research on solid-state spin qubits has revealed that, in addition to the magnetic spin bath, charge defects constitute a primary source of decoherence (*17*, *25*). While existing studies have largely focused on monitoring and spatially localizing charge fluctuations in the dc-megahertz regime at cryogenic temperatures (*19*) or suppressing noise via electric field depletion control (*17*, *25*, *26*), a method for broadband and species-resolved identification of nanoscale noise sources remains unrealized. This gap hinders the optimization of material growth and device fabrication processes for both the semiconductor industry and quantum technologies. Here, we bridge this gap by developing a room-temperature, broadband (near-dc to gigahertz) quantum noise spectrometer using individual PL5 centers in commercial 4H-SiC. Empowered by the extreme electric field sensitivity and nanoscale resolution, we capture real-time charge dynamics. Expanding from this microscopic insight to a macroscopic metrology tool, we achieve frequency-resolved characterization of the noise environment—from mapping the heterogeneities and quality between different wafers to obtaining the nanoscale electron paramagnetic resonance (EPR) fingerprint of spin defects hosted in the material. Last, by comparing experimental results before and after surface treatment, we identify the main physical origin of the noise as the native SiO_2_ layer on the surface.

## RESULTS

### Experimental setup and sensing scheme

**Fig. 1.**
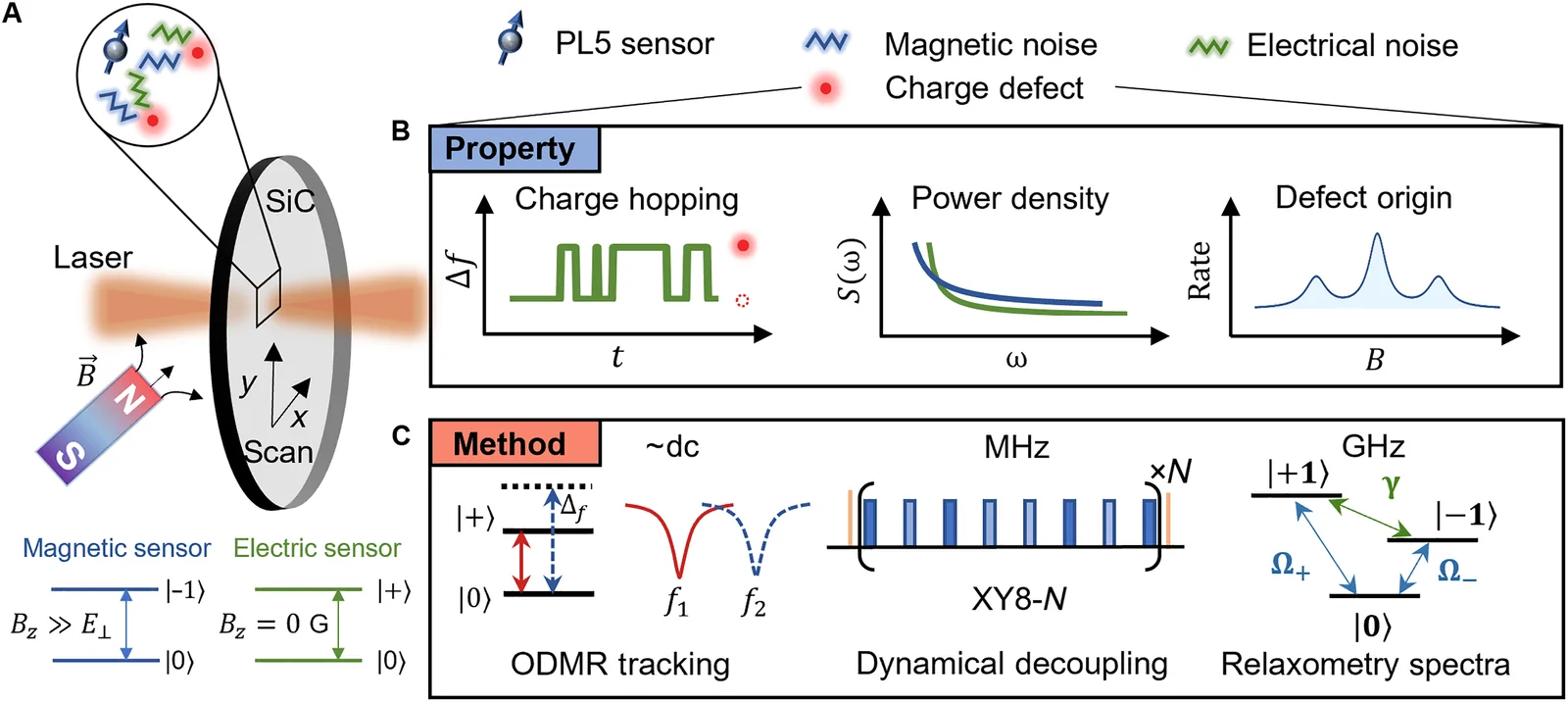
Experimental setup and sensing scheme overview. (**A**) Experiment principle of quantum sensor in silicon carbide. A 905-nm laser is used to realize the initialization and readout of PL5 center. Electrical and magnetic noise in semiconductors can have a significant impact on the performance of high-power electronic devices and on the coherence properties of qubits embedded in solid-state materials. An external magnetic field parallel to the principal axis of PL5 center is applied to tune the energy levels when needed. The intrinsic none-zero term $E_{x}$ for PL5 makes the transition between ground states {∣0〉,∣+〉} exclusively sensitive to transverse electric noise under zero magnetic field. When $B_{z}$ is applied, the transition become sensitive to longitudinal magnetic noise. (**B**) Physical properties of the local charge environment. The panels illustrate three key dimensions of defect characterization: (left) charge hopping, showing the time-domain random telegraph noise induced by discrete charge trapping and releasing events; (middle) power density, displaying the frequency-domain noise spectrum for both magnetic (blue) and electrical (green) noise components; and (right) defect species, revealing the spectroscopic fingerprint used to identify specific defects. (**C**) Corresponding quantum sensing protocols. The methods span from dc to gigahertz frequencies: (left) real-time tracking of ODMR resonance shifts Δf monitor low-frequency fluctuations; (middle) dynamical decoupling sequences (e.g., XY8) with variable interpulse spacing to probe noise power density in the megahertz regime; and (right) $T_{1}$ relaxometry–based EPR spectroscopy to probe charge defect species in the gigahertz regime.

A full landscape of our broadband noise detection technique routine in silicon carbide at room temperature is shown in Fig. 1. Our sample was diced from a wafer consisting of an intrinsic epitaxial layer of single-crystal 4H-SiC grown on a 4° off-axis N-type 4H-SiC substrate. To generate PL5 centers, we implanted 60-keV ^14^N^+^ ions at a dose of 10^10^ cm^−2^, followed by annealing for 30 min. A 905-nm laser is used to initialize and read out the spin state of a single PL5 quantum sensor in 4H-SiC, where a fluorescence count rate of 250 kcps for a single PL5 center has been observed without photonic structure enhancement. The Hamiltonian of PL5 center is given by

$$H/h=\frac{1}{\hbar^{2}}\left[DS_{z}^{2}-E_{x}\left(S_{x}^{2}-S_{y}^{2}\right)+\gamma B\cdot S\right]$$ (1)

where D≈1360 MHz and $E_{x}\approx 16\;\mathrm{MHz}$ (the coordinate is chosen to parallel the *c* axis to the *xz* plane) (*22*, *23*). The Hamiltonian dictates that under zero magnetic field, the nonzero term $E_{x}$ makes the transition between ground states exclusively sensitive to transverse electric noise. Microwave pulses are applied to coherently manipulate the spin. An external magnetic field parallel to the principal axis of PL5 center (labeled as $B_{z}$) is applied to tune the energy levels when needed. As illustrated in Fig. 1 (B and C), we use a suite of coherent control sequences—ranging from continuous-wave (CW) monitoring to dynamical decoupling and $T_{1}$ relaxometry—to filter and track spectral fluctuations across a wide frequency bandwidth (near-dc to gigahertz). This setup effectively converts the PL5 center into a localized broadband spectrum analyzer for the surrounding electrical and magnetic environment induced by charge defects.

### Electrometry of two charge traps

**Fig. 2.**
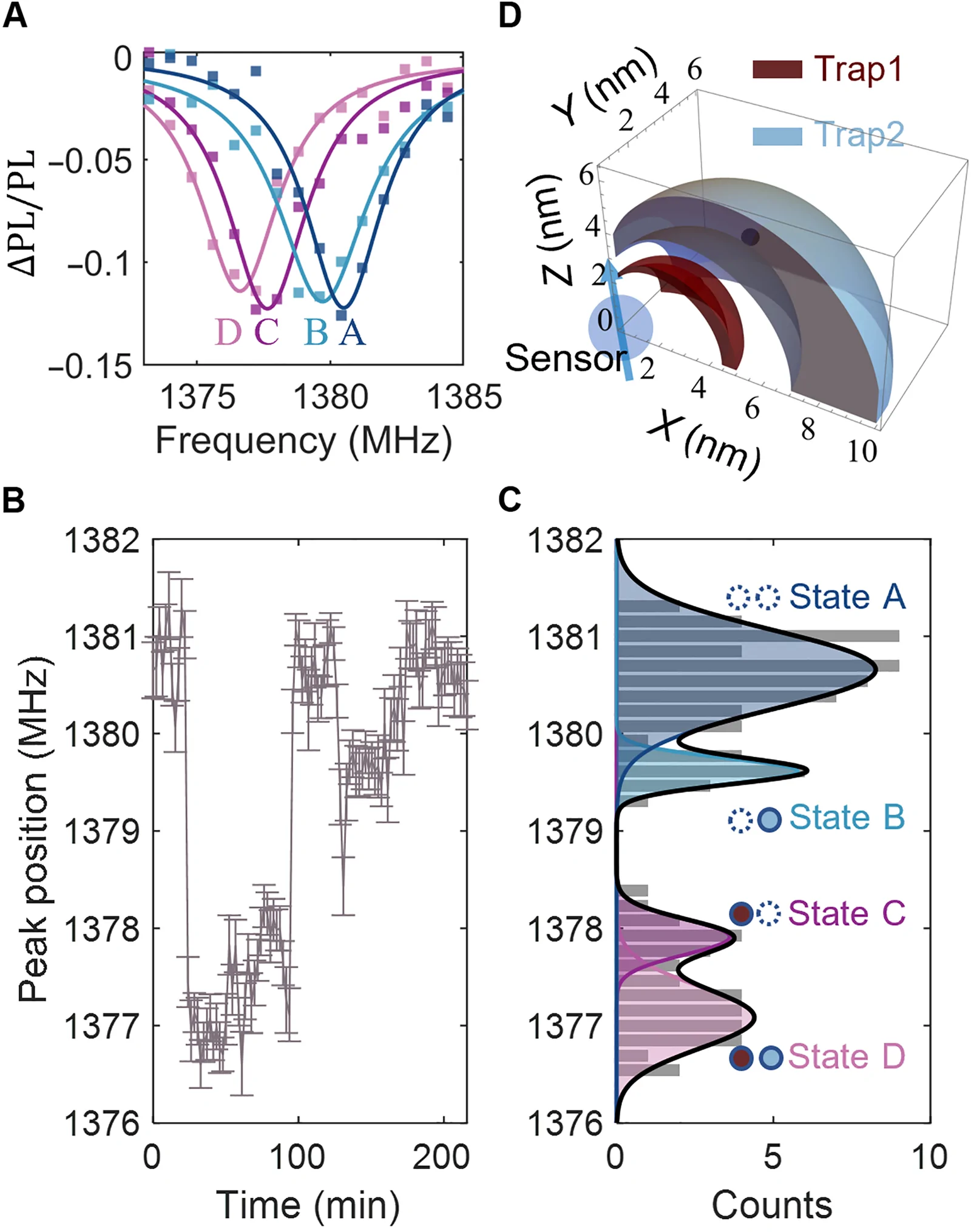
Nanoscale monitoring of electric environment at single charge level. (**A**) Four typical CW spectral configurations with different central frequencies of a single quantum sensor were observed, which were labeled as A to D. (**B**) Time-resolved CW-ODMR measurements on a single PL5 center reveal the evolution of its spectral peak position. (**C**) Histogram illustrates the distribution of peak positions in (B). Four well-defined stable states can be clearly identified by fitting, corresponding to the four states identified in (A). The peak separation between states A and C (B and D) is 3 ± 0.3 MHz, while that between states A and B (C and D) is 1 ± 0.3 MHz. This indicates that the observed spectral fluctuations result from a composition of two distinct two-level system signals. (**D**) The possible locations of the two charge traps with 95% confidence intervals based on the splitting of different states shown in (C). The detail of the calculation can be found in section S2.3 of the Supplementary Materials.

The change of local electric field will cause Stark shift between ∣0〉 and ∣+〉/∣−〉 transitions as displayed in Fig. 1C, where $|+\rangle /|-\rangle =\frac{1}{\sqrt{2}}\left(|+1\rangle \pm |-1\rangle \right)$. Because of the orientation of the microwave field, spin manipulation between the ∣0〉 and ∣+〉 is more effective under zero magnetic field in our setup. For certain PL5 centers, we observed time-dependent spectral switching in their CW–optically detected magnetic resonance (ODMR) results as shown in Fig. 2. Figure 2A presents four representative ODMR spectral configurations (labeled state A to state D) acquired from the same individual color center. The raw data and their corresponding Lorentzian fits show that the sensor exhibits distinct resonance frequencies with shifted central frequencies at different moments, indicating a dynamic local environment. To systematically capture these dynamics, we performed a continuous spectral tracking measurement over a duration of several hours. The resulting time-resolved map, shown in Fig. 2B, visualizes the temporal evolution of the peak positions. By compiling the resonance frequencies extracted from this long-term trace, we obtained the statistical distribution presented in Fig. 2C. We attribute this behavior to the surrounding traps, which cause fluctuation of electric field when they capture or release charges. On the basis of the spectral splitting between different states (3 ± 0.3 MHz between states A and C and 1 ± 0.3 MHz between states C and D), we constrained the possible locations of the two charge traps with 95% confidence interval using the Stark shift coefficients of PL5 centers. Given that the transverse Stark coefficient is substantially larger than its longitudinal counterpart [$d_{⊥}=32.5,d_{∥}<3\;\mathrm{Hz}\cdot \mathrm{cm}/V$ ] (*24*), we infer that the observed electric field fluctuation originates from charge trapping and releasing events of two traps within a region of less than 10 nm (Fig. 2D). More analysis is shown in section S2 of the Supplementary Materials.

### Noise imaging and material characterization

**Fig. 3.**
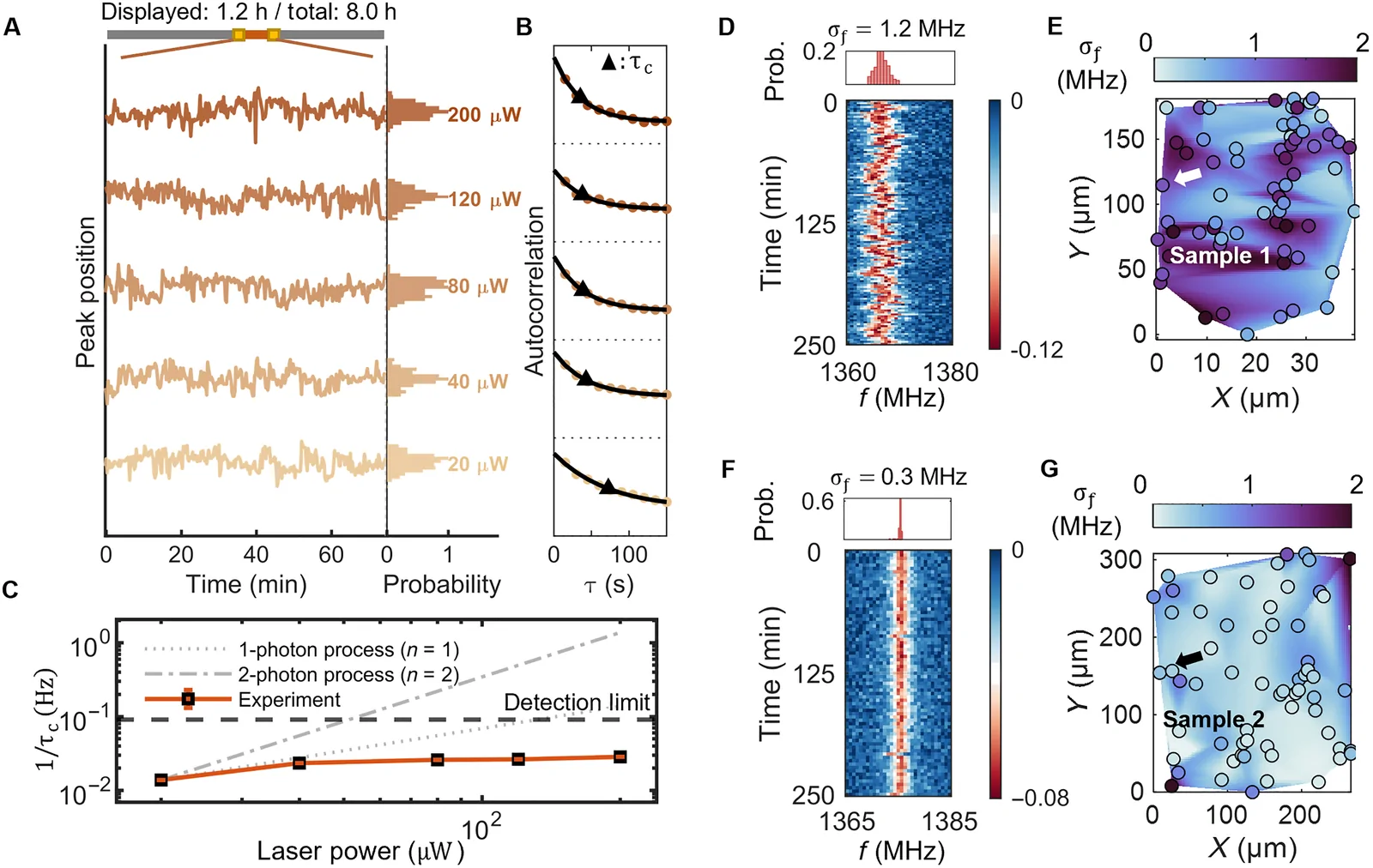
Continuous charge jitter and nanoscale electrical noise imaging of commercial SiC wafers. (**A**) Long-term CW ODMR tracking of a representative PL5 sensor under different laser powers. The sensor shows a random, continuous spectral wandering, which represents the typical noise behavior for most investigated defects. For visual clarity, only a portion of the data and its distribution at each laser power is displayed. (**B**) ACF of the spectral trajectories. Solid black lines are exponential fits used to extract the characteristic fluctuation time constant ($\tau_{c}$). Details of the ACF extraction and analysis are provided in the Supplementary Materials. (**C**) Extracted fluctuation rate ($1/\tau_{c}$) as a function of laser power. Even as the optical power spans an order of magnitude, the fluctuation rate exhibits only a marginal increase, remaining consistently far below the theoretical rates expected for single- or two-photon processes. This result indicates that this continuous noise is driven by a thermally activated background bath rather than direct optical excitation. The fit error bars (1σ) are smaller than the size of the data markers. (**D**) Spectral diffusion tracking and the corresponding probability distribution of a typical sensor from wafer 1 (sample 1), showing a broad peak distribution ($\sigma_{f}=1.2\;\mathrm{MHz}$). (**E**) Nanoscale electrical noise map of wafer 1. The spatial distribution of σ_f_ reveals clear variations in the local charge environment over tens of microns. Colored maps are interpolated from the measured (σ_f_) values at individual PL5 locations; circles mark the measured sensors. (**F** and **G**) Corresponding spectral tracking and noise map for wafer 2 (sample 2). Despite having the same fabrication history, wafer 2 shows a much higher spectral stability ($\sigma_{f}=0.3\;\mathrm{MHz}$). This contrast demonstrates the use of our quantum probe to evaluate intrinsic variations in wafer quality. h, hours.

While the charge hopping events observed in Fig. 2 provide a microscopic picture of single–charge defect dynamics, this discrete jumping behavior is quite rare, appearing in less than 1% of the hundreds of PL5 sensors we investigated. For the vast majority of sensors, the spectral instability appeared as quasi-continuous jitter where individual charge transition events are hard to be clearly resolved. To systematically quantify this noise, we performed long-term CW-ODMR tracking on these representative unstable sensors. As shown in Fig. 3A, the peak position exhibits continuous and stochastic wandering over several hours. To distinguish the underlying physical mechanism, we extracted the fluctuation rates using autocorrelation function (ACF) analysis across various laser excitation powers (Fig. 3B). When the optical power changes by one order of magnitude, the increase in the fluctuation rate remains less than threefold (Fig. 3C), implying that these fluctuations are more likely driven by a thermally activated background bath rather than single/two-photon processes. More analysis and data are provided in sections S2.5 to S2.6 of the Supplementary Materials. To evaluate the applicability of our method for material characterization, we investigated two 4H-SiC epitaxial wafers (wafer 1 and wafer 2) obtained from different commercial manufacturers. Both samples were processed using identical ion implantation and annealing protocols to generate the PL5 center probes. Despite sharing the same ion implantation process, the sensors exhibited notably different noise environments. While wafer 1 showed widespread spectral diffusion and instability (Fig. 3D), wafer 2 demonstrated high spectral stability with minimal near-dc electrical noise (Fig. 3F). This pronounced contrast demonstrates that our method is sufficiently sensitive to reveal the intrinsic differences in charge trap density and material quality between two wafers. By correlating the spatial locations of individual sensors with their measured noise levels, quantified by the standard deviation of the ODMR peak positions ($\sigma_{f}$), we constructed electrical noise maps for both samples (Fig. 3, E and G). The noise map of wafer 1 reveals macroscopic spatial variations in noise density over tens of micrometers, and the distribution of fluctuation rate across different PL5 sensors extracted by the ACF method is provided in section S2.5 of the Supplementary Materials. This demonstrates the potential of our method as a powerful characterization tool for imaging nanoscale electric noise distributions with single-charge sensitivity and wafer-quality diagnostics. Furthermore, conventional electrical characterization and ensemble defect measurements on both wafers yield results consistent with the disparities observed via our quantum sensors (section S4 of the Supplementary Materials). This agreement supports the reliability of the PL5-based measurements, while the single-defect quantum probes provide complementary nanoscale insight into local charge-noise heterogeneity beyond the wafer-averaged response.

### Noise spectrum and EPR identification

**Fig. 4.**
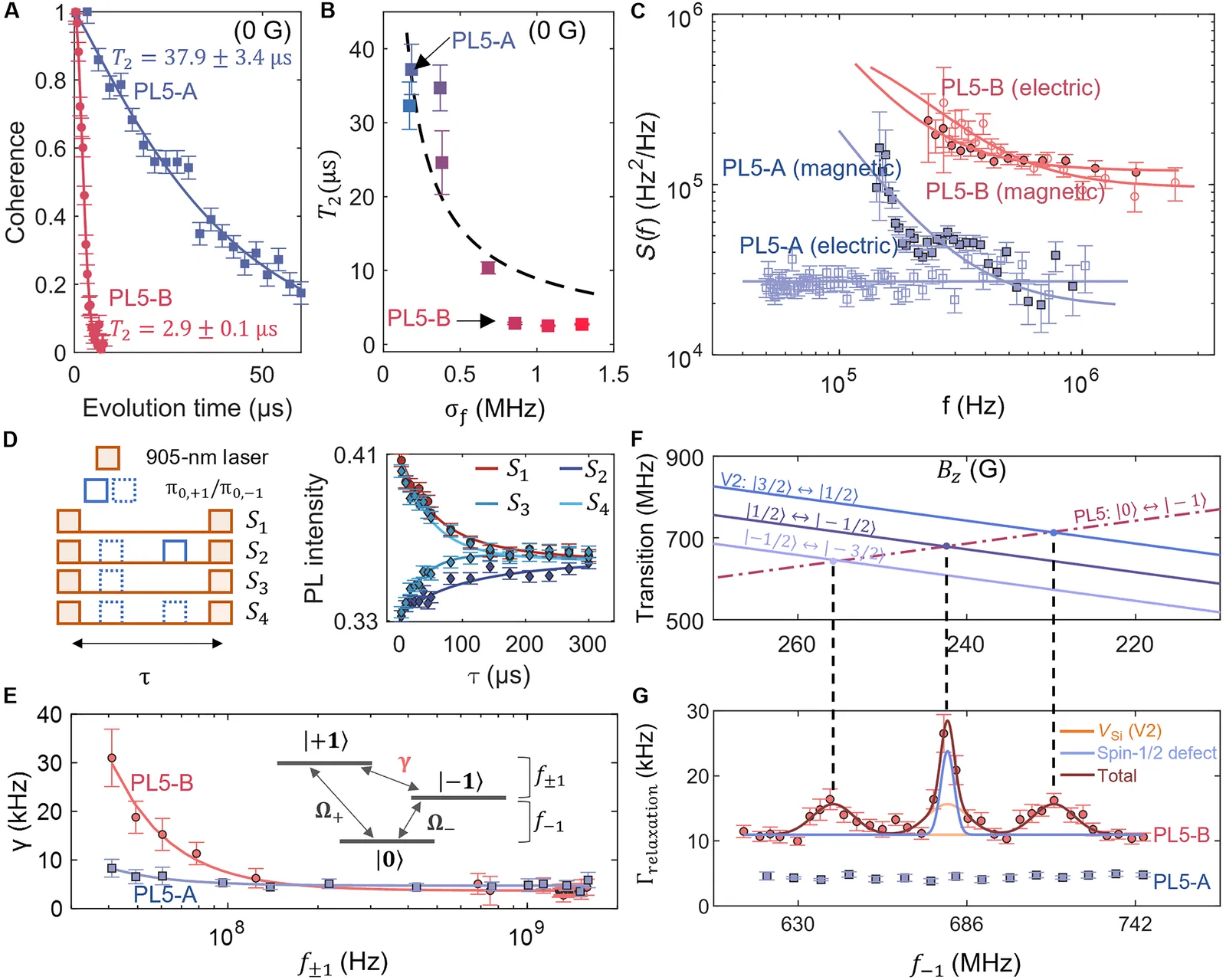
Noise strength correlation and spectrum of PL5 centers with EPR identification of noise source. (**A**) Hahn echo $T_{2}$ measurement of the two PL5 centers (named PL5-A and PL5-B) on wafer 1. The sensor with lower near-dc noise (PL5-A) exhibits a longer coherence time. (**B**) A rapid decrease in Hahn echo coherence time is observed as the spectral fluctuation ($\sigma_{f}$) increases, indicating a strong correlation between near-dc noise and high-frequency electrical noise. The dashed curve serves as a fitting guideline with $T_{2}\propto 1/{\sigma_{f}}^{k}$. (**C**) Noise spectra obtained from XY8 dynamical decoupling measurements on PL5-A and PL5-B. Experiments were performed under both zero field and 120-G magnetic field aligned along the defect axis, fitted with Lorentzian model. (**D**) SQ and DQ transition rates are extracted using different spin state initialization and readout schemes. Right panel shows the measurement result of PL5-B at 260.4 G along the PL5 axis. Fitting model follows the equation shown in Eq. 2. (**E**) Comparison of electrical noise strength γ between the two PL5 centers, showing that the unstable sensor exhibits a stronger 1/f-type noise. (**F**) Transition energies (Δ) for PL5 center and V2 center are plotted against magnetic field. (**G**) $T_{1}$ relaxometry for two PL5 centers. Here, relaxation rate Γ was extracted from *S*_1_ to *S*_3_ shown in (D). The observed accelerated relaxation of PL5-B reveals the presence of environmental noise. On the basis of the extracted spectral peak position and calculation of transition energy shown in (F), we attribute this signal to nearby silicon vacancy (V2) and other spin-1/2 traps, while no signal was observed for PL5-A. Error bar is given by 1 SD.

Having visualized the spatial heterogeneity of the electric noise, we carried out a detailed study on sample 1 in subsequent experiments. We sought to identify its microscopic origin and explore the correlation in frequency domain. First, we measured the Hahn echo coherence time $T_{2}$ of eight sensors. Among these, two representative PL5 centers (designated PL5-A and PL5-B) exhibiting different CW-ODMR stability ($\sigma_{fA}\;=\;0.18\;\mathrm{MHz}$ and $\sigma_{fB}\;=\;0.86\;\mathrm{MHz}$) were selected for detailed investigation. Note that, here, we use the two-point measurement method to enhance the temporal resolution (*27*, *28*). As shown in Fig. 4A, the unstable sensor PL5-B exhibits a pronounced reduction in $T_{2}$ by up to an order of magnitude compared to the stable sensor PL5-A. To more clearly reveal this correlation, we plotted σ_f_ of the eight sensors against their Hahn echo coherence times as shown in Fig. 4B.The observed trend indicates that PL5 centers experiencing stronger near-dc spectral diffusion generally exhibit shorter Hahn echo coherence times. The dashed line is fitted with $T_{2}\propto 1/{\sigma_{f}}^{k}$ (k≈0.8), an empirical power law fit to the sensors under the assumption of a universal noise spectral density across the sample. However, for the three PL5 centers with the largest values of $\sigma_{f}$, the measured coherence times fall below the fitted trend. These experimental deviations suggest that the local defect environments around these PL5 centers may exhibit distinct dynamical behaviors. To further investigate the noise spectra of PL5-A and PL5-B at higher frequencies up to megahertz, we implemented XY8-1 dynamical decoupling protocols on the two PL5 under zero-field and axial magnetic field (∼120 G), with the resulting noise spectrum analyzed using Lorentzian fits (*29*). At zero magnetic field, longitudinal magnetic noise is suppressed by the transverse strain field $E_{x}$, so decoherence is governed by electric noise along *x* direction. In contrast, under $B_{z}>$ 100 G, the transverse electromagnetic fluctuations are suppressed, and because the Stark coefficient for the longitudinal electric response of PL5 center is exceptionally small, longitudinal magnetic noise becomes the dominant source of decoherence (*29*, *30*). Under the δ function filter approximation, we obtained the noise spectrum as shown in Fig. 4C. The results indicate a difference in the noise environments of the two sensors. For the spectrally stable PL5-A at near-dc range (with lower $\sigma_{f}$), the electric noise spectrum also stays at a low level in the submegahertz range, implying that its magnetic noise is predominantly caused by the surrounding spin bath. In contrast, PL5-B exhibits significantly higher electric noise, accompanied by a magnetic noise intensity several times greater than that of PL5-A. This suggests that, beyond the intrinsic spin bath, PL5-B is coupled to local spin-active or paramagnetic fluctuators whose charge-state dynamics are correlated with the enhanced electric noise environment. These findings confirm that minimizing the proximity effect of electromagnetic noise through fundamental charge engineering is a critical step to realizing high-coherence quantum sensors (*17*, *25*). More results of higher-order dynamical decoupling are provided in section S3.1 of the Supplementary Materials.

To extend our noise spectroscopy window from the megahertz to gigahertz regime, we applied a magnetic field along the PL5 center axis and scanned $B_{z}$ to tune the PL5 centers energy levels and subsequently performed $T_{1}$ relaxation measurements. By measuring the evolution curves under different initialization and readout conditions $S_{1}$ to $S_{4}$ as shown in Fig. 4D, we extracted the single-quantum (SQ) and double-quantum (DQ) transition rates, denoted as $\Omega_{+}/\Omega_{-}$ and γ as shown in Fig. 4E (inset) (*29*), which represent the magnetic and electric noise strength in our system. After preparing the initial state of PL5 center at the eigenstates $m_{s}=0,\pm 1$ by laser and microwave pulses ($\pi_{0,+1},\pi_{0,-1}$), the process of longitudinal relaxation of spin state ρ(t) can be described by rate equation

$$\dot{\rho }\left(t\right)=\Gamma \rho \left(t\right)$$ (2)

where the decay matrix is given by (*29*, *31*)

$$\Gamma =\left(\begin{array}{ccc} -\Omega_{+1}-\gamma & \Omega_{+1} & \gamma \\ \Omega_{+1} & -\Omega_{+1}-\Omega_{-1} & \Omega_{-1}\\ \gamma & \Omega_{-1} & -\Omega_{-1}-\gamma \end{array}\right)$$ (3)

On the basis of this method, we observed two important phenomena. First, in the tens-of-megahertz regime of DQ transition rate measurements, we observed $1/f^{\alpha }$-type electrical noise in both PL5-A and PL5-B, as shown in Fig. 4E. The PL5-B, which shows unstable ODMR positions in former experiment, exhibits stronger $1/f^{\alpha }$ noise compared to PL5-A. This confirms the presence of an ensemble of charge fluctuators in the environment surrounding the unstable quantum sensor, which provide valuable insight into the characterization of the noise environment in semiconductors. Second, we examined its magnetic noise spectrum by varying the magnetic field and extracted relaxation rate Γ from *S*_1_ to *S*_3_ shown in Fig. 4D. The resulting spectra reveal a direct causal link between specific defect species and charge noise. For PL5-B, we observed distinct resonance peaks at *g* = 2.0, accompanied by satellite features (Fig. 4, F and G), which can be attributed to relaxation enhancement between PL5-B and nearby traps. Data were fitted using a composite model comprising three Gaussian functions of equal amplitude and width, supplemented by an additional central Gaussian peak. On the basis of the fitting result ($f_{1}=641.58\pm 2.24\;\text{MHz},f_{2}=679.66\pm 0.28\;\text{MHz},\text{and}\;f_{3}=714.94\pm 2.24\;\text{MHz}$) and theoretical calculation (Fig. 4F), our results corresponds to a defect configuration consisting of silicon vacancies [*V*_Si_(V2), 2*D* = 70 MHz] (*32*), while the distinct relaxation peak emerging in the middle provides a signature of other spin-1/2 dark traps background (e.g., carbon vacancies *V*_C_), as shown in Fig. 4G. In comparison, we observed no signal for the CW-stable quantum sensor PL5-A. When the $B_{z}$ was scanned over a wide range from 20 to 300 G, the SQ and DQ transition rates remained unchanged, maintaining a ratio $\frac{\gamma }{\Omega }$ ≈ 2.4, where $\Omega \;\equiv \;\Omega_{+1}\;=\;\Omega_{-1}$ (see section S3.1 of the Supplementary Materials). This result demonstrates the capability of our method to perform spatially resolved chemical identification. By retrieving spectroscopic fingerprints from distinct locations, we can effectively resolve variations in the local spin environment within different nanoscopic regions of the sample using a single quantum sensor. Last, our estimation of the magnetic sensing range (5 to 8 nm) shows a spatial consistency with the electrical noise measurements, and more results on different PL5 centers are provided in section S2.7 of the Supplementary Materials.

### Native oxide noise origin revealed by surface engineering

**Fig. 5.**
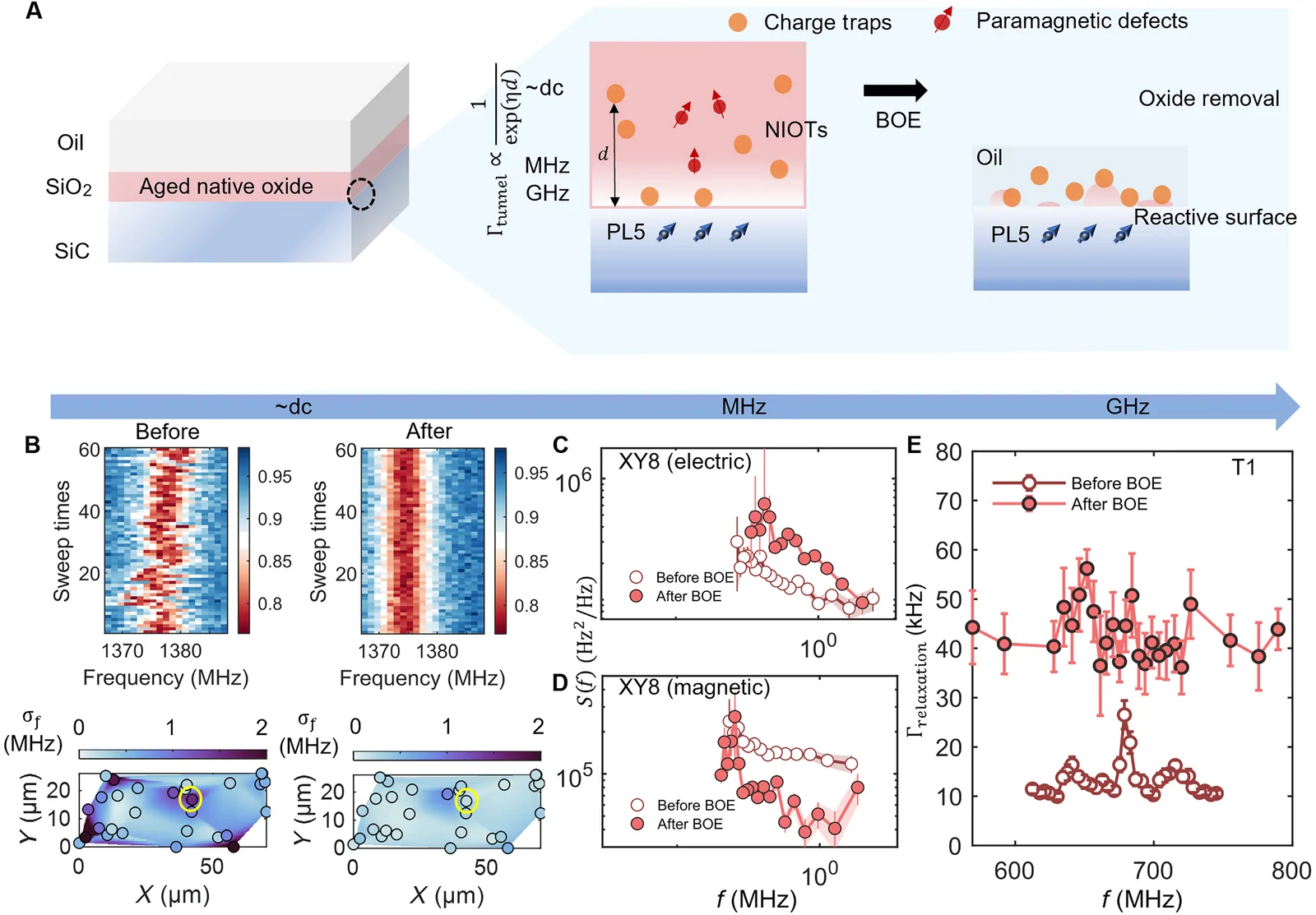
Validation of the interface spectral noise model via surface chemical engineering. (**A**) Schematic of the SiC-SiO_2_ interface architecture. The charge tunneling rate $\left(\Gamma_{\text{tunnel}}\right)$ is exponentially dependent on the trap depth *d*. BOE strips the aged native oxide and its near-dc noise contribution of NIOTs but exposes a reactive bare SiC surface that rapidly accumulates unpassivated surface states (e.g., dangling bonds). (**B**) Near-dc noise stabilization. Continuous ODMR tracking (top) and corresponding spatial noise maps (bottom) show a global suppression of the slow spectral diffusion (σ_f_) after BOE treatment in sample 1. (**C** to **E**) Comparison of broadband noise decoupling results and $T_{1}$ collapse for the unstable sensor PL5-B. In the megahertz regime, the electric noise at 0 G (**C**) increases due to the highly active bare surface (more charge traps), whereas the magnetic noise at 120 G (**D**) decreases because of the removal of the oxide paramagnetic spin bath. In the gigahertz regime (E), the $T_{1}$ relaxation rate ($\Gamma_{\text{relaxation}}$) increases significantly, obscuring the previously resolved EPR resonances.

To establish a physical microscopic origin for the observed broadband noise, we consider the architecture of the SiC-SiO_2_ interface. Extensive semiconductor device studies indicate that the oxide inherently hosts a substoichiometric SiO*_x_* transition layer (*33*) populated with near-interface oxide traps (NIOTs). Crucially, the charge tunneling dynamics of these defects are exponentially dependent on their physical distance from the semiconductor surface, generating a massive hierarchy of fluctuation timescales from nanoseconds to hours (*34*). Such a temporal hierarchy can span from slow near-dc drift to nanosecond-scale fluctuations, making local broadband noise spectroscopy particularly important for understanding the heterogeneous SiC/SiO_2_ interface quality and its impact on device-relevant noise. Here, we performed surface engineering using a buffered oxide etch (BOE, 7:1) to chemically strip the aged native oxide layer (Fig. 5A). This intervention fundamentally reconfigures the local defect landscape through a dual effect: It physically removes the thick outer oxide, while simultaneously exposing a highly reactive SiC surface that rapidly develops unpassivated, dynamic surface states and oxide islands upon brief exposure to the ambient environment. This reconfiguration results in a frequency-dependent decoupling of the quantum noise detected by the PL5 sensors. By stripping the thick native oxide, we effectively suppress the slow and intermediate noise channels. In the near-dc regime, spatial ODMR-tracking maps show a systematic reduction of slow spectral diffusion (σ_*f*_) after BOE treatment, while a representative PL5 trace in Fig. 5B illustrates this stabilization at the single-sensor level. To examine how this surface modification affects different frequency channels in the same local environment, we further performed a detailed before/after comparison on the previously studied unstable sensor PL5-B. For PL5-B, $\sigma_{f}$ decreases from 0.86 to 0.20 MHz. Similarly, in the intermediate megahertz regime, the magnetic noise extracted at 120 G is significantly suppressed (Fig. 5D), with an increase in the XY8 coherence time from 7.16 ± 0.63 to 12 ± 0.61 μs. This 1.76-fold enhancement indicates the quenching of the paramagnetic spin bath associated with the aged oxide. However, the newly exposed surface also introduces strong charge fluctuations, which elevate the high-frequency electric noise channel and are accompanied by a pronounced gigahertz magnetic noise background, likely arising from newly formed paramagnetic fluctuators near the reactive surface. Consequently, the overall $T_{1}$ relaxation rate ($\Gamma_{\text{relaxation}}$) exhibits a substantially increased background (Fig. 5E), obscuring the previously resolved EPR resonance signals. Ultimately, these divergent spectral trends illustrate how the structural depth of interfacial defects dictates the footprint of quantum decoherence. Details of the surface treatment procedure and additional before/after comparisons for other PL5 centers are provided in sections S2.7 and S3.2 of the Supplementary Materials.

## DISCUSSION

In summary, our study establishes a robust, broadband quantum sensing protocol capable of diagnosing wafer-level quality variations with nanoscale precision using individual PL5 centers as room-temperature noise spectrometers. Through systematic monitoring, we demonstrate real-time observation of single-charge tunneling dynamics at room temperature in silicon carbide. Expanding from this microscopic insight, we introduce a spatial imaging approach for noise characterization that provides a quantitative local indicator of wafer quality. The difference between wafers resolved by PL5 probes is further supported by conventional macroscopic characterization techniques, including CW-EPR, *I-V/C-V*, mercury-probe *C-V*, and DLTS, as detailed in section S4 of the Supplementary Materials. These measurements provide wafer-level benchmarks that are consistent with the PL5 observations and help place the local quantum-sensing results in a broader material characterization context. Beyond mapping the noise, our broadband spectroscopy provides a frequency-resolved view of the local noise channels contributing to decoherence. By integrating dynamical decoupling and $T_{1}$ relaxometry, we provide the nanoscale EPR spectroscopic fingerprint of charge defects in SiC. The identification of distinct local spin species, such as silicon vacancies (V2) and other spin-1/2 defects, demonstrates dipole-dipole interactions among heterogeneous qubits (PL5 and V2). Given their distinct excitation and emission bands (*35*, *36*), this highlights the promising prospect of realizing scalable hybrid quantum registers within the same material platform. Furthermore, by combining broadband sensing with targeted surface chemical treatment (BOE), we identify a major contribution of the native oxide layer to the measured broadband noise. The before/after comparison supports a depth-dependent spectral picture: Removing the oxide suppresses slow near-dc spectral diffusion and intermediate megahertz magnetic noise from structurally constrained traps but simultaneously exposes a highly reactive bare SiC surface that drives ultrafast charge fluctuations and a massive gigahertz magnetic noise background. Overall, individual quantum probes provide nanoscale access to local interfacial noise, enabling single–charge level dynamics and frequency-resolved electric/magnetic noise spectroscopy from near-dc to gigahertz regimes. This local, broadband perspective complements wafer-scale characterization by linking macroscopic material quality variations to microscopic noise dynamics.

From the perspective of qubit optimization, our results highlight the role of substrate-hosted and interfacial charge environment in driving solid-state qubit decoherence. By identifying the frequency domains in which these fluctuators contribute, this work emphasizes the importance of high-quality substrates and controlled surface passivation for realizing high-performance and scalable quantum systems.

From the perspective of advanced metrology, our work demonstrates the capability of shallow PL5 centers as quantum probes for local interface diagnostics. Combined with near-infrared excitation, which minimizes above-bandgap photocarrier generation in 4H-SiC, this approach complements diamond NV-based sensing methods, especially in situations where external electric field sensing can be affected by surface charge screening. Looking forward, commercial SiC wafers may be developed into smart quantum substrates or scanning probes for local multimodal diagnostics. Such a platform could be useful for monitoring diverse nanoscale environments, ranging from ferroelectric vortices in epitaxially grown van der Waals heterostructures (*37*) to interface Coulomb scattering in SiC-CMOS devices (*38*).

This approach can be generalized to other defect species in SiC and wide-field imaging can be incorporated in practical measurements to increase the speed and field of view (*13*). This method is expected to serve as a vital tool for the precise characterization of third-generation semiconductors, paving the way for high-throughput, atomic-scale defect engineering and spin-spin interfaces of qubits implemented in silicon carbide for quantum information and sensing applications.

## MATERIALS AND METHODS

### Optical setup

A home-built scanning confocal microscope equipped with a three-axis piezoelectric stage (CoreMorrow Ltd., P15.XYZ300S-B) and a high–numerical aperture (NA = 1.45) infrared oil-immersion objective (Nikon, CFI Plan Apochromat Lambda D) was used in the experiments. Excitation of the PL5 was achieved using a 905-nm laser source (Changchun New Industries, MDL-III-905-SM). The fluorescence emission was further filtered through a longpass filter and then coupled into a single-mode fiber, guided to a superconducting nanowire single-photon detector from Photon Technology (P-SPD-8S). Photon counts were recorded using a data acquisition counter (JYTEK, PCIe-5510).

### Coherent manipulation setup

Microwave pulses were generated by a microwave signal generator (Rigol, DSG-3065B), followed by gating via a high-speed microwave switch (Mini-Circuits, ZASWA-2-50DR+). The signals were subsequently amplified using a broadband microwave amplifier (Mini-Circuits, ZHL-25 W-272+) and delivered to a gold coplanar waveguide fabricated beneath the surface of the 4H-SiC sample. Synchronization of the optical pulses, microwave signals, and photon counting was precisely controlled by a digital delay-pulse generator from CIQTEK.

### Sample information

In this study, quantum sensors were investigated in two samples, designated sample 1 and sample 2. The two samples are from two different wafers purchased from different companies. Sample 1 was prepared by cutting from a wafer comprising a 12.5-μm-thick low-doped epitaxial layer of single-crystal 4H-SiC grown on a 4° off-axis N-type 4H-SiC substrate. Similarly, sample 2 was obtained from a wafer featuring a 30-μm-thick low-doped epitaxial layer of single-crystal 4H-SiC grown on a 4° off-axis N-type 4H-SiC substrate. Both samples underwent ion implantation with 60-keV ^14^N^+^ ions at a fluence of ∼1 × 10^10^ cm^−2^. Postimplantation annealing was performed in a vacuum furnace at 1000°C for sample 1 and 1050°C for sample 2, each for a duration of 30 min. Crucially, while typical intrinsic defects (e.g., divacancies and PL1 to PL4) generated by the 60-keV implantation reside deeper in the bulk, the PL5 color centers investigated here are structurally oxygen vacancy (*23*, *39*). To evaluate their depth profile, we performed Stopping and Range of Ions in Matter simulations (*40*), modeling the process with an assumed 1-nm-thick native oxide (SiO_2_) layer on the SiC surface. As illustrated in fig. S1, the primary intrinsic vacancies (Si and C vacancies) exhibit a broad distribution peaking ∼80 to 100 nm below the surface. In contrast, the oxygen atoms recoiled from the native oxide layer are distributed significantly shallower, predominantly confined to the top few nanometers. Although the subsequent high-temperature annealing process may induce some spatial redistribution and defect migration, this initial recoil profile strongly suggests that the oxygen vacancy–related PL5 complexes are fundamentally shallow quantum sensors in our 60-keV ^14^N^+^ ion implantation, making them exquisitely sensitive to the silicon carbide interfacial environment.

## Supplementary Materials

### This PDF file includes:

Sections S1 to S4 Figs. S1 to S17 Table S1 References
