# Supplementary material for: Quantum noise spectroscopy of nanoscale charge defects in silicon carbide at room temperature
Source: Sci Adv. 2026 Sep 25;12(39):eaej5462. doi: 10.1126/sciadv.aej5462 (PMC13614393; doi:10.1126/sciadv.aej5462)
Supplement: Supplementary file 1 [file sciadv.aej5462_sm.pdf]

Science Advances
AAAS

Supplementary Materials for

Quantum noise spectroscopy of nanoscale charge defects in silicon carbide at room temperature

Jinpeng Liu *et al.*

Corresponding author: Qi Zhang, zhq2011@ustc.edu.cn; Fazhan Shi, fzshi@ustc.edu.cn

Sci. Adv. **12**, eaej5462 (2026)
DOI: 10.1126/sciadv.aej5462

This PDF file includes:

Sections S1 to S4
Figs. S1 to S17
Table S1
References

S1. SUPPLEMENTARY SAMPLE CHARACTERIZATION AND DATA OVERVIEW

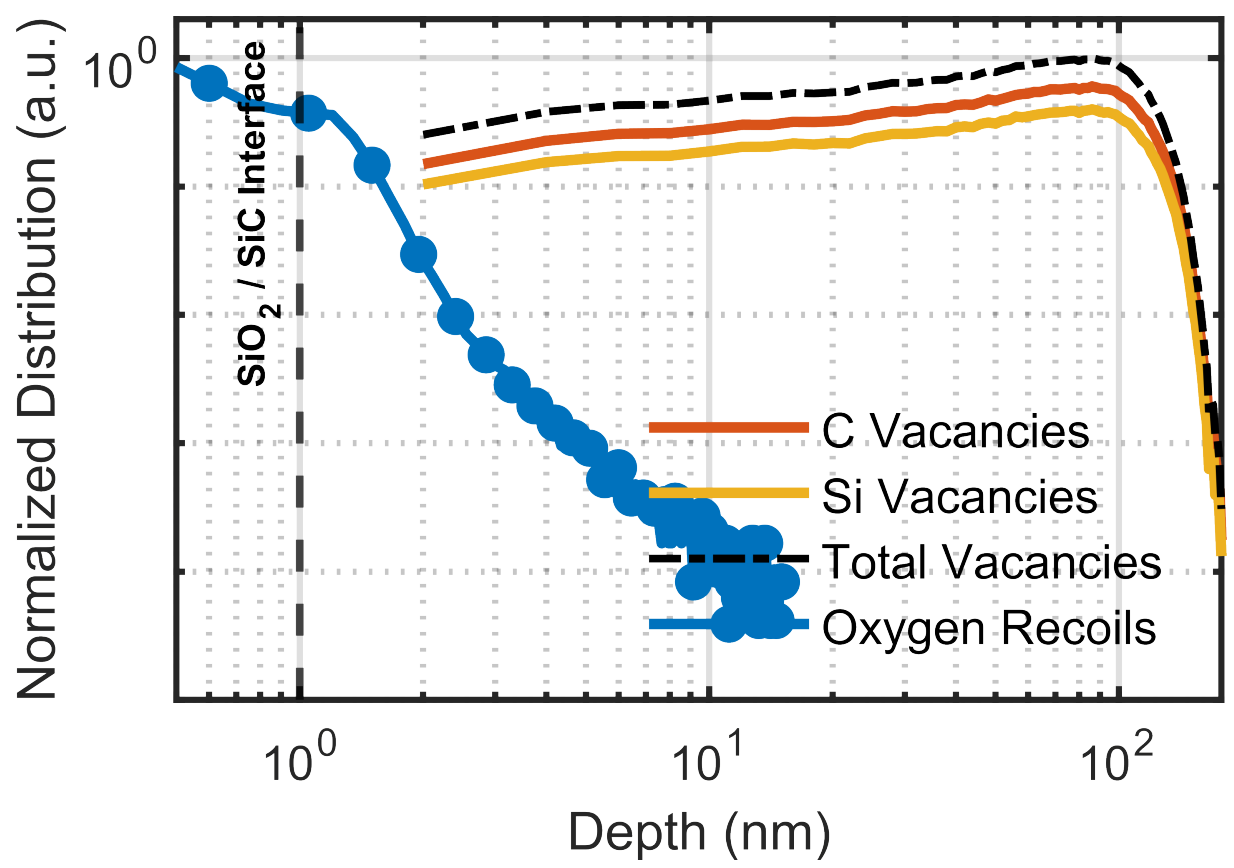

FIG. S1. Normalized depth profiles of oxygen recoils (blue circles) and lattice vacancies in 4H-SiC following a 60 keV $^{14}N^+$ ion implantation. The simulation assumes a 1-nm-thick native SiO_2 layer on the SiC surface. Unlike the intrinsic vacancies which peak deep in the epi layer (100nm), the oxygen recoils exhibit a drastically shallower spatial distribution. This difference confirms the near-surface nature of the oxygen-related PL5 color centers in our samples.

To provide a comprehensive overview of the quantum sensors investigated in this work and to clarify the statistical support for our claims, we summarize the PL5 centers used across different experimental figures in Table SI. Besides, to verify the generation of single color centers using our current fabrication recipe, we characterized the sample using confocal photoluminescence microscopy. As shown in Fig. S2(a), the confocal map reveals discrete fluorescent spots with a well-distributed brightness across the scanning area. To confirm the nature of these emitters, we performed a second-order photon autocorrelation measurement on a representative spot exhibiting typical PL5 brightness, as marked by the orange circle. The resulting curve displays a pronounced anti-bunching dip with $g^{(2)}(0) = 0.15$, unambiguously demonstrating that the isolated spots generated under our current recipe are single color centers.

TABLE SI: **Summary of PL5 color centers investigated in this study.** This table outlines the specific color centers (identified by defect number, No.), the corresponding wafers, and the experiments performed to provide a clear mapping of the data presented in the main text figures.

Experiment	Wafer (1/2)	Color center (PL5)
Single charge tracking (Fig.2)	1	No.1634
Laser power dependency (Fig.3(a-c), Fig.S9)	1	No.328, 988
DC noise statistics and mapping (Fig.3(d-g))	1 & 2	64 color centers in Sample 1<br>63 color centers in Sample 2;<br>Representative traces:<br>No.24 in Sample 1, No.141 in Sample 2
T_2 (Hahn echo) (Fig.4(b))	1	8 color centers (No.24, 120, 178<br>363, 385 (PL5-B), 423, 453 (PL5-A), 487)
DD & EPR relaxometry<br>(Fig.4(a, c-g), Fig.5(c-e))	1	No.453 (PL5-A)<br>No.385 (PL5-B)
DC noise comparison<br>before/after BOE (Fig.5b)	1	30 color centers in Sample 1<br>Representative traces: No.202

S2. ANALYSIS OF ODMR TRACKING

S2.1. HMM Model and PCA Clustering

The behavior of dark defects capturing and releasing charges can be modeled using a hidden Markov model (HMM), with the capture and emission processes characterized by time constants $\tau_{release}$ and $\tau_{capture}$ respectively [41, 42]. In complex charge environments, it is often difficult to directly infer the number of hidden states from the spectral peak positions alone. For these color centers, fluctuations in the local electric field often occur within the timescale of a single CW-ODMR scan, leading to broadened or distorted spectral peaks, and in some cases, the appearance of multiple peaks. To better identify and classify these hidden spectral states, we treated each CW contrast curve as a feature vector and applied a clustering algorithm [43, 44]. Principal component analysis (PCA) was used to reduce dimensionality and visualize the classification results, as shown in Figure S3(a). The optimal number of hidden states was determined using the silhouette coefficient, which indicates that

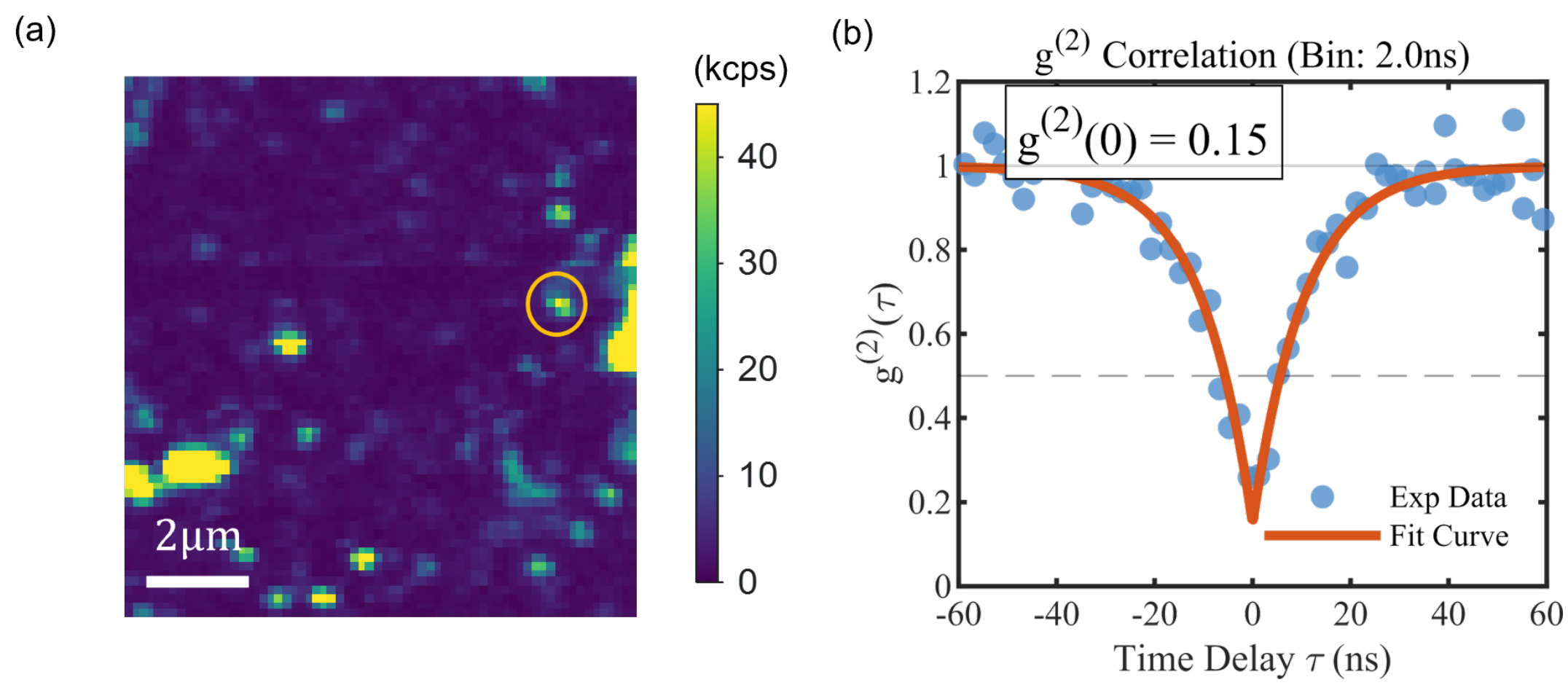

FIG. S2. (a) Confocal fluorescence image of the SiC sample, showing discrete fluorescent spots with good brightness uniformity. The orange circle indicates a representative color center selected for autocorrelation analysis. (b) Second-order autocorrelation $g^{(2)}(\tau)$ measurement of the circled spot in (a). The pronounced anti-bunching dip at zero delay, $g^{(2)}(0) = 0.15 < 0.5$, confirms that the emitter is a single color center

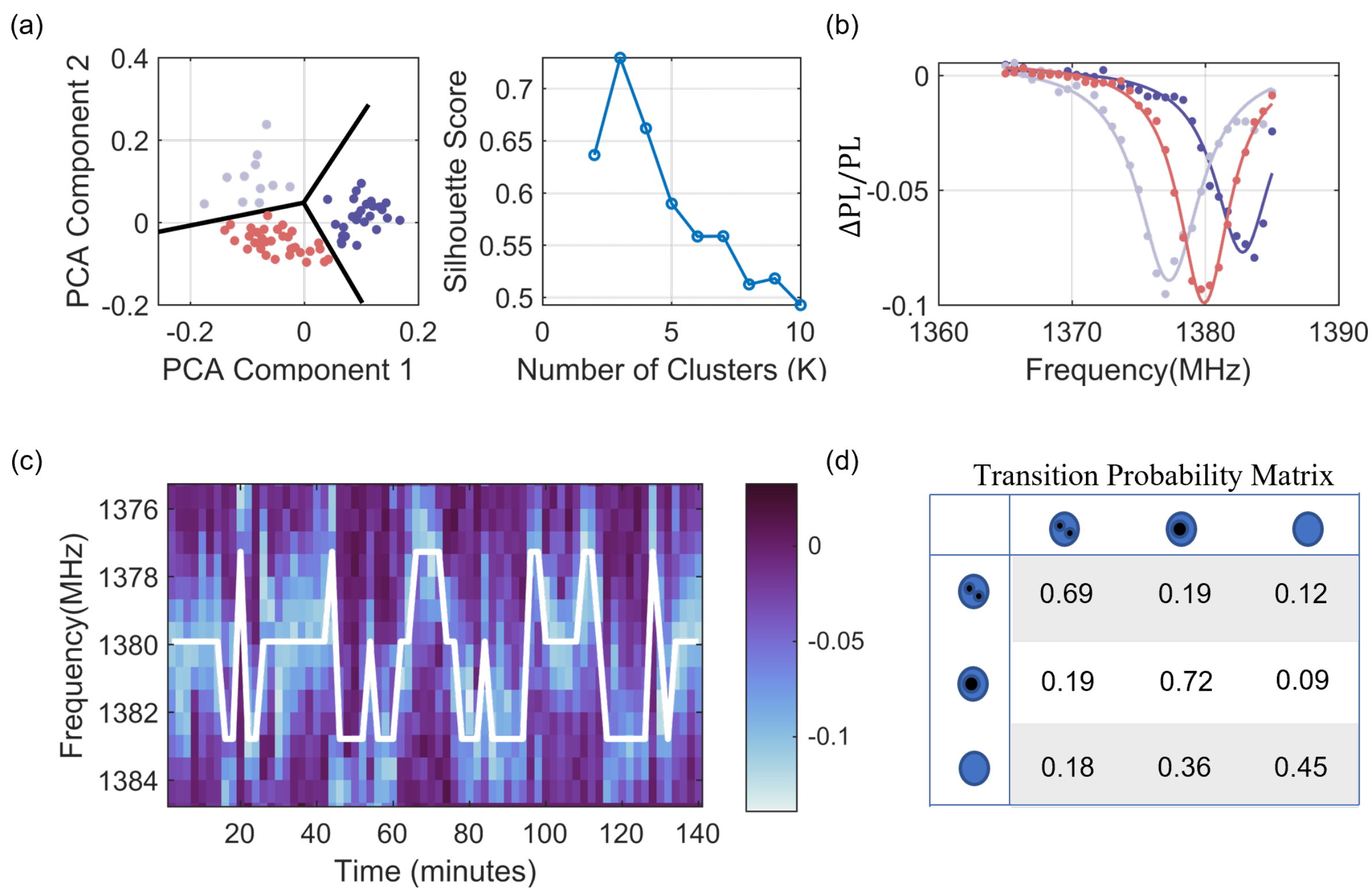

FIG. S3. (a) Clustering results visualized via principal component analysis (PCA), based on feature vectors constructed from contrast values at different frequencies. Silhouette coefficient is plotted as a function of cluster number, indicating optimal clustering performance at three clusters (K=3), where inter-class separability is maximized and intra-class variance is minimized. (b) Averaged CW spectral lineshapes corresponding to each of the three identified states. (c) Experimental CW spectral data and a three-state Markov process model based on the clustering result above. (d) Extracted transition constants for each state from the hidden Markov model, suggesting that the three discrete states may originate from surrounding dark defects with different charge configurations.

three clusters provide the best separation for this dataset. The average contrast curves for each cluster are shown in Figure S3(b). We attribute the three observed states to different charge configurations of nearby dark charges. The transition rates between these states can be extracted using HMM analysis, as shown in Figure S3(c-d).

S2.2. CW Tracking between two color center

To rule out the influence of hardware delays or other experimental effects on spectral stability measurements, we selected two color centers with distinct stability characteristics, labeled OV_a and OV_b. We performed repeated CW-ODMR measurements switching between the two centers. As shown in Figure S4, the results confirm that the observed spectral peak shifts are not caused by measurement errors but truly exists.

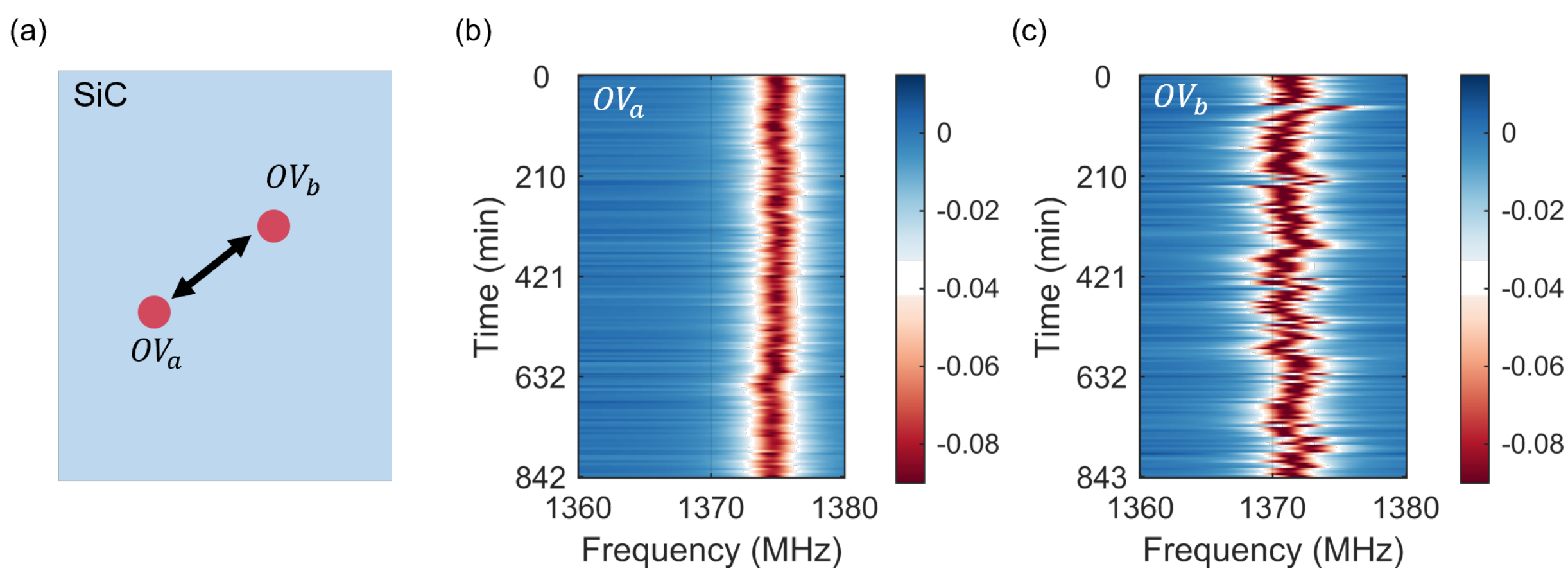

FIG. S4. (a) Schematic of the experiment. We continuously alternate between two color centers, performing a CW-ODMR scan at the position of color center OV_a to obtain the spectrum, then immediately move to color center OV_b for measurement, before returning to OV_a. This process repeats in a cycle. (b-c) Results obtained by separating the experimental data from the two color centers. A clear distinction is observed between the stable and unstable centers, demonstrating the reliability of the signals we observed.

S2.3. Long-Time ODMR Stability Tracking

In this case, we performed repeated, long-duration measurements on one of the unstable color centers. Over time, the previously observed two-level signal evolves into a mixed behavior involving both ensemble-level spectral drift and discrete spectral jumps [Figure S5 (a)]. To analyze this behavior, we apply a differential treatment to the long-term data, as

shown in Figure S5(b), where the y axis represents the peak shift between adjacent measurements (Δf). A Gaussian fit reveals large amount of small shift (purple data) together with outlier points beyond 3σ (orange data). As illustrated in Figure S5(c) and (d), we attribute these deviations to a combination of contributions from a broader ensemble of distant charge defects (~10 nm range) and a smaller number of nearby charges located within ~5 nm of the color center PL5. Figure S5(d) is a numerical result of the relation between a single trap position and the right peak position of PL5 caused by stark shift, where we assume $d_{\perp}$ =32.5(Hz cm/V) and the trap lies within the same plane as the stress axis of PL5, since the Stark response along the z-direction is weak [24]. The x-axis is defined along the direction of the local stress field $\vec{E}$ (17.2MHz) of the color center. The calculation result shown in the main text Fig.2(d) was similar except that we set $d_{\parallel} = 1$(Hz cm/V).

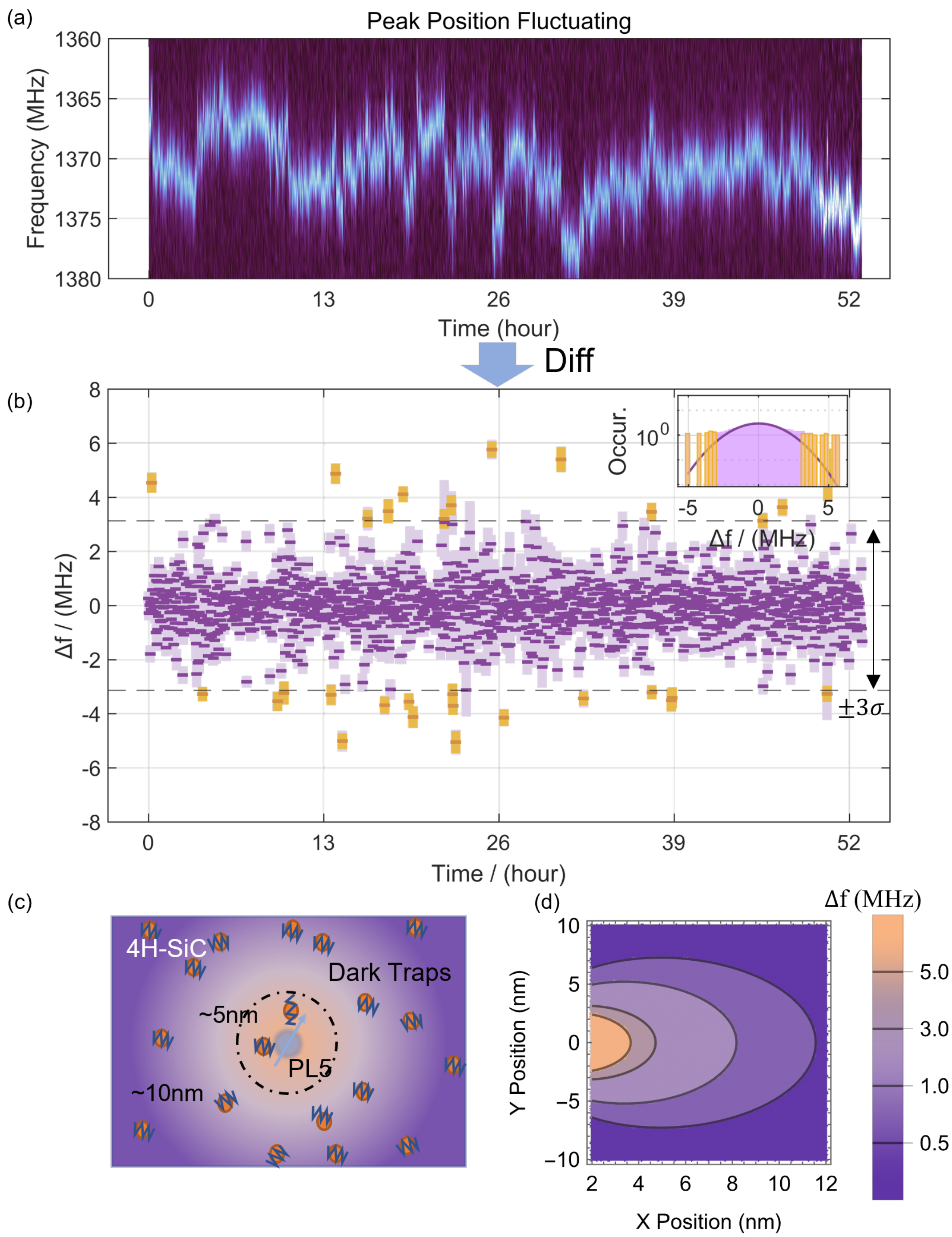

FIG. S5. (a) CW spectral stability of a single color center monitored over tens of hours. The spectral peak exhibits two-state-like switching behavior together with a long-term gradual drift. (b) The fitted peak positions were numerically differentiated. Points falling outside the ($\pm 3\sigma$) region are identified as discrete charge-jump events nearby, while the underlying drift is attributed to background noise from the ensemble charge environment. (c) Electrical noise environment around PL5. Under this model, the observed spectral peak shift is contributed by two parts: the electric field drift from distant ensemble defects and the charge release and capture from nearby single trap. After long-duration experiments, the properties of the nearby traps may change and stabilize, leading to an increased contribution from the ensemble drift, and making the two level signal indistinguishable. (d) Relation between the charge location and corresponding stark shift Δ_f, where here we assume the charge is located in the plane of E_x.

S2.4. ACF Analysis

As described in Section S2.2 and Section S2.3, high-signal-to-noise ratio (SNR) data resolving discrete single-charge dynamics is rare. In most experimental scenarios, color centers are subject to ensemble-like electronic noise from the background environment, which obscures the distinct switching between discrete states using HMM method and clustering. To overcome this, we choose to employ Autocorrelation Function (ACF) analysis to robustly extract the underlying state fluctuation rate Γ.

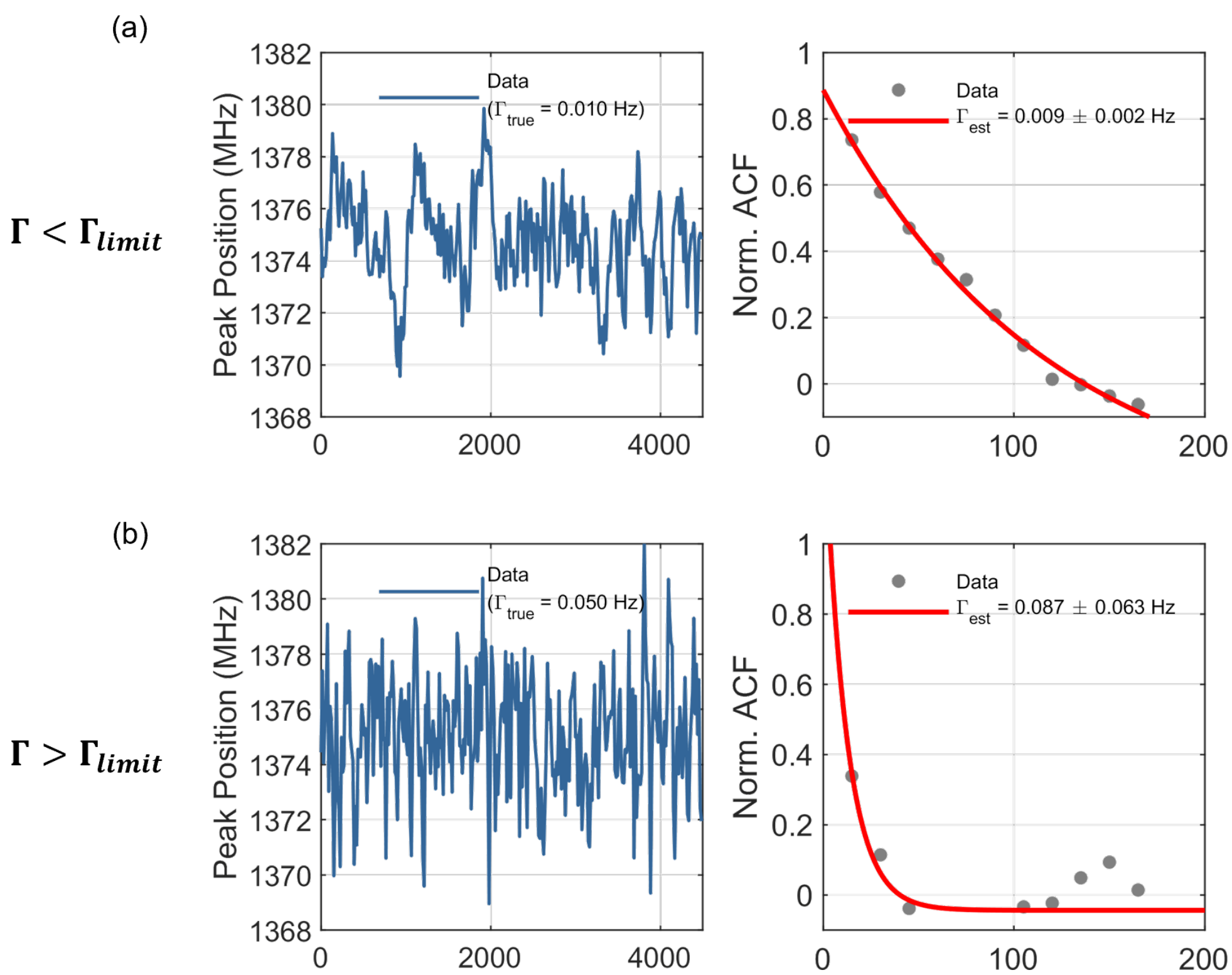

FIG. S6. (a-b) Left panel: A synthetic dataset ($N = 300$, $dt = 15$ s) generated using an Ornstein–Uhlenbeck process to simulate spectral peak jitter. The model incorporates two different ground-truth transition rate Γ_{true} , overlaid with white noise arising from measurement precision and ensemble charge noise. The extracted ACF (gray circles) are derived from the simulation time series. The red solid line represents a nonlinear least-squares fit to the exponential decay model $A \cdot \exp(-\Gamma\tau) + t$. The extracted rate $\Gamma_{est} = 0.009 \pm 0.002$ Hz (1σ fit uncertainty) for $\Gamma_{true} = 0.01$Hz, which shows excellent agreement with the pre-set physical value, confirming the accuracy of the methodology for experimental data analysis. While for another situation shown in (b) where $\Gamma > \Gamma_{limit}$, the fit is failed with a large error $\Gamma_{est} = 0.087 \pm 0.063$ Hz.

To validate the reliability of this approach, we first generated a simulated time series that mimics experimental peak-position jitter under typical noise conditions around 1375 MHz. This allows us to demonstrate the algorithm's capability to recover the physical transition rate even when the raw signal is embedded in significant white noise and baseline drift as

shown in Fig. S6. The red solid line represents a nonlinear least-squares fit to the exponential decay model $A \cdot \exp(-\Gamma\tau) + t$. Simulation results demonstrate that exponential fitting can accurately resolve transition rates for dynamics slower than our detection limit. Furthermore, for processes exceeding this limit, we can distinguish between them by analyzing the fitting state and the associated fitting errors.

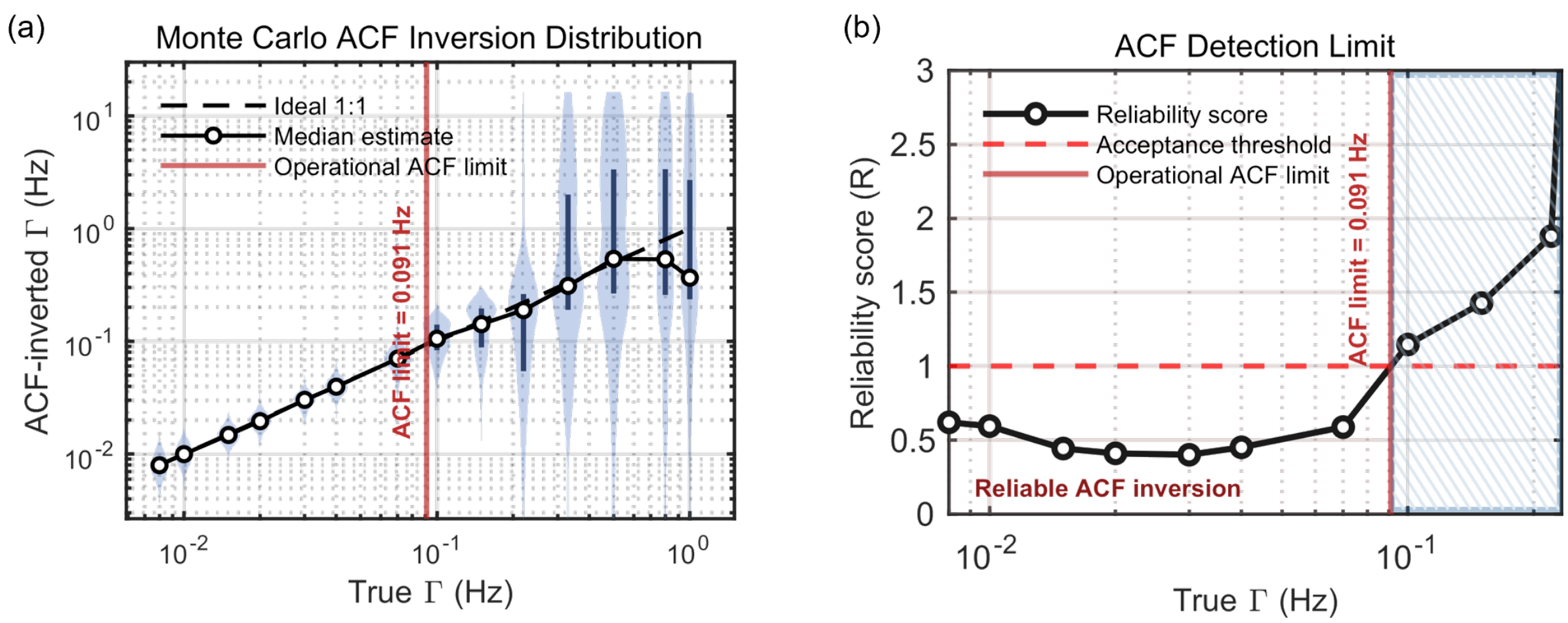

FIG. S7. (a) For each prescribed relaxation rate Γ_{true}, 300 synthetic peak-position trajectories were generated using an Ornstein–Uhlenbeck process with the same sampling interval, trajectory length, fluctuation amplitude, and white-noise level as in the experiment. The violin plots show the distribution of ACF-extracted rates Γ_{ACF}, while the black circles indicate the median values. The dashed line denotes the ideal relation $\Gamma_{\text{ACF}} = \Gamma_{\text{true}}$. (b) The score R combines the relative median bias and the normalized interquartile range of the Monte Carlo-extracted rates. The ACF inversion is considered reliable for $R < 1$, corresponding to a median bias below 30% and an IQR below 50% of Γ_{true}. The red dashed horizontal line indicates the acceptance threshold $R = 1$, and the red vertical line gives the resulting operational detection limit of approximately 0.091 Hz. Beyond this limit, the ACF fit can still return finite rates, but the extracted values are no longer statistically stable.

To quantify the practical detection limit of the ACF analysis (Γ_{limit}), we performed Monte Carlo simulations using the same acquisition parameters as in the experiment. The simulated peak-position trajectories were generated from an Ornstein–Uhlenbeck process with a prescribed relaxation rate Γ_{true}, a stationary fluctuation amplitude of 2.2 MHz, and an additional white measurement noise of 0.5 MHz. Each trajectory contained $N = 2000$ points sampled every $\Delta t = 15$ s. For each value of Γ_{true}, 300 independent trajectories were generated and analyzed using the same ACF fitting procedure as that used for the experimental data.

The extracted rate distributions broaden as Γ_{true} approaches the temporal resolution limit of the ACF method. We therefore define an operational ACF detection limit using both accuracy and precision criteria. Specifically, the ACF inversion is considered reliable

only when the relative median bias is below 30% and the interquartile range (IQR) of the extracted rates is below 50% of Γ_{true}. These two conditions are combined into a reliability score R we defined as:

$$R = \max\left(\frac{|\text{median}(\Gamma_{\text{ACF}}) - \Gamma_{\text{true}}|/\Gamma_{\text{true}}}{0.30}, \frac{\text{IQR}(\Gamma_{\text{ACF}})/\Gamma_{\text{true}}}{0.50}\right). \tag{S1}$$

We define condition $R < 1$ as the reliable regime in our experiment. Under the present experimental sampling condition discussed above, this criterion gives an operational ACF detection limit of approximately 0.091 Hz. Rates above this value may still yield finite fitted values, but the recovered distribution becomes sufficiently broad that the result should no longer be interpreted as a quantitatively reliable ACF inversion.

S2.5. Distribution of Fluctuation Rate across Sensors

We performed ACF analysis on the CW stability data of 64 color centers in Sample 1. By filtering for centers with a fitting coefficient $R^2 > 0.7$, we retained 44 sensors to generate the statistical spatial and rate distributions shown in Fig. S8. The results indicate that the charge fluctuations in the sample are predominantly associated with slow processes in the frequency range of 0.02 to 0.1 Hz. Faster fluctuations could not be resolved using the ACF method in this experiment.

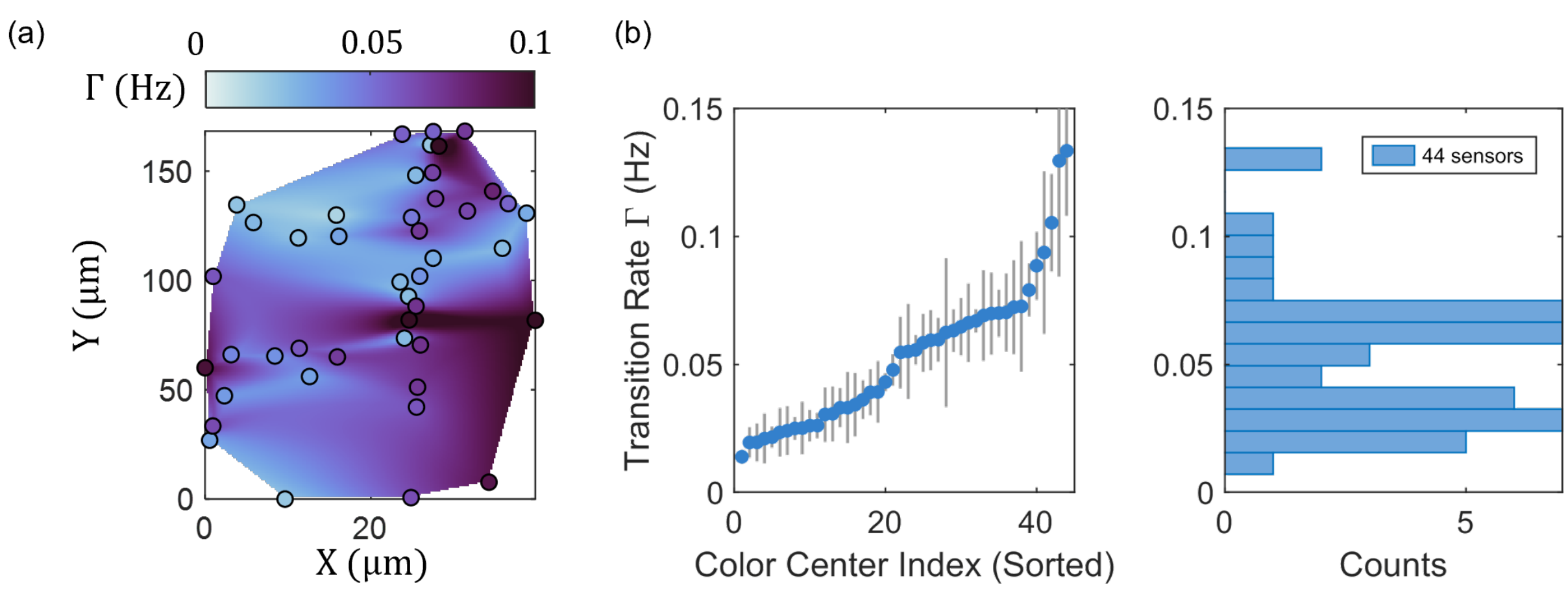

FIG. S8. Statistical distribution of charge fluctuation rates in Sample 1. (a) Spatial map of the ACF-extracted transition rates (Γ) for the 44 reliable color center sensors (Fitting $R^2 > 0.7$). (b) Histogram of the corresponding transition rates. The distribution reveals that the local charge environment is dominated by slow dynamic processes, concentrated in the range of 0.02 to 0.1 Hz

Besides, we observed this broad distribution with two apparent characteristic scales around 0.025 and 0.06 Hz. Given the limited statistics, we do not assign a specific microscopic origin. Nevertheless, this result indicates substantial heterogeneity in the local fluctuator environments.

S2.6. Laser Power Dependency of CW Stability

To examine whether the observed CW jitter is predominantly governed by optical excitation, we next performed laser-power-dependent measurements on two representative PL5 centers selected from the characteristic rate ranges identified in the statistical ACF distribution above. Using the ACF method described above, we extracted the characteristic time scales of the color centers at different laser powers; the reciprocal value $1/\tau_c$ gives the transition rate Γ. The two selected PL5 centers correspond to typical local dynamics within the observed distribution Fig. S8(b), including the No.328 sensor presented in the main text. As shown in Fig. S9, the extracted transition rates show only weak dependence on 905 nm laser power. This behavior is far weaker than expected for a dominant one- or two-photon-driven process. When the optical power changes by one order of magnitude, the increase in the transition rate remains less than threefold. Therefore, while we cannot exclude weak photo-assisted perturbations for rare individual defects, the combined statistical and power-dependent results indicate that the dominant near-DC charge instability observed in Sample 1 is not predominantly governed by optical excitation.

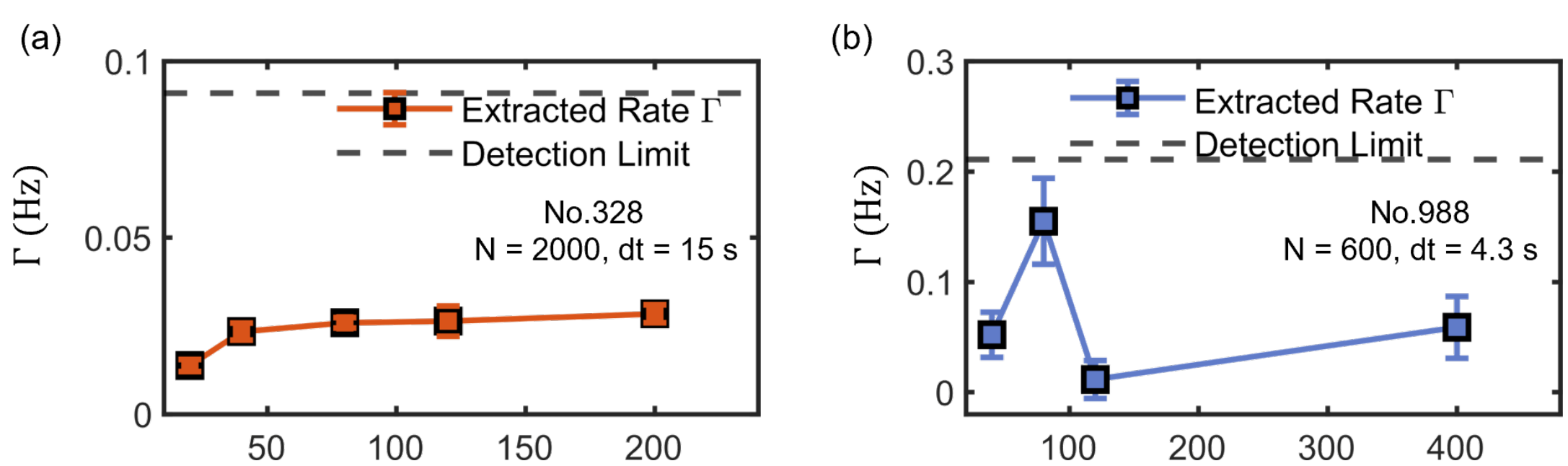

FIG. S9. Power-independent stability of color center dynamics. The ACF-extracted transition rates for different color centers, plotted as a function of optical excitation power. The data indicate that the transition rates exhibit minimal sensitivity or only slightly increase with power variations over an order of magnitude. The SN328 center is consistent with the results presented in the main text. The detection limit is determined according to different sampling parameters N and dt.

S2.7. Correlation with Magnetic Sensing Range

To provide more evidence that the electrical and magnetic noise sources share a common origin, we analyze the sensing range of the T_1 relaxation spectroscopy and compare it with the defect distribution derived from the Stark shift analysis (Figure S5(d)). The cross-relaxation (flip-flop) interaction between the sensor and a dark spin leads to a resonant reduction in the measured T_1 time when their transition frequencies match. Solving the Lindbald equation, the decay rates induced by dark electrons can be expressed as

$$Q = \frac{4\gamma^2 \Gamma_D}{\Gamma_D^2 + \Delta^2} \tag{S2}$$

where we assume the dark eletron has similar T_1 decay rates with PL5 center ($\sim$ 10kHz) and Γ_D can be esitimated via the FWHM of spectrum shown in Figure S10(a). The sensitivity of our relaxation measurement is given by

$$\delta_\Gamma = \frac{2}{\tau \exp(-\tau/T_d)} \frac{1}{\sqrt{N}} \frac{1}{C\sqrt{\alpha}} \tag{S3}$$

, τ is the evolution time, T_d is the T_1 decay rate of PL5 center and C, α represent the contrast and photon number in a single measurement, N is the experiment cycles in one sec. By calculating the For a system consisting of S=1 and S=1/2, the effective coupling strength is given by the magnetic dipole-dipole interaction:

$$\gamma = \frac{1}{2\sqrt{2}} \frac{\mu_0 g_e^2 \mu_B^2}{4\pi\hbar r^3} \left|1 - 3\cos^2\theta\right| \tag{S4}$$

where r is the distance and θ is the angle with respect to the quantization axis (z-axis). Figure S10(b) shows the calculated sensing boundary when SNR=1. The maximum sensing radius extends to approximately $5 \sim 8$ nm.

Remarkably, this magnetic sensing range aligns well with the electrical charge noise range estimated from the long-term ODMR spectral diffusion shown in Figure S5(d). This spatial consistency strongly suggests that the fluctuating charges responsible for the Stark shifts and the dark spins causing T_1 relaxation likely originate from the same environment in the vicinity of the sensor. To systematically validate the impact of the BOE treatment on the local spin environment (e.g.,dangling bond), we statistically analyzed the T_1 relaxation rates (Γ) of 8 individual color centers (including PL5-A and PL5-B). As shown in Fig. S10(c), measurements were performed at two specific magnetic fields: 222 G (off-resonance, representing the broadband high-frequency magnetic background noise) and 242 G (on-resonance,

corresponding to the g = 2.0 paramagnetic defects), both before and after BOE treatment. The statistical results yield two key observations. First, the baseline relaxation rates at 222 G (light blue bars) near-universally increase post-BOE across all measured centers. This indicates that the etching process introduces intense, higher-frequency magnetic noise at the newly exposed interface, elevating the overall relaxation background. Second, the relative signal strength of the paramagnetic defects—represented by the difference in relaxation rates between 242 G and 222 G ($\Delta\Gamma = \Gamma_{242} - \Gamma_{222}$)—exhibits an decrease after the BOE treatment for some PL5 (typically, Defect 2, 5 and 7). This suggests a reduction in the density of these specific structural paramagnetic defects upon the removal of the oxide layer. Notably, the variation in T_1 and the relative signal strength exhibit distinct differences among individual color centers (inset figure). This implies the complexity of the interfacial spin environment and highlights the heterogeneity of the local nanoscale semiconductor interface experienced by different color centers, which warrants further investigation.

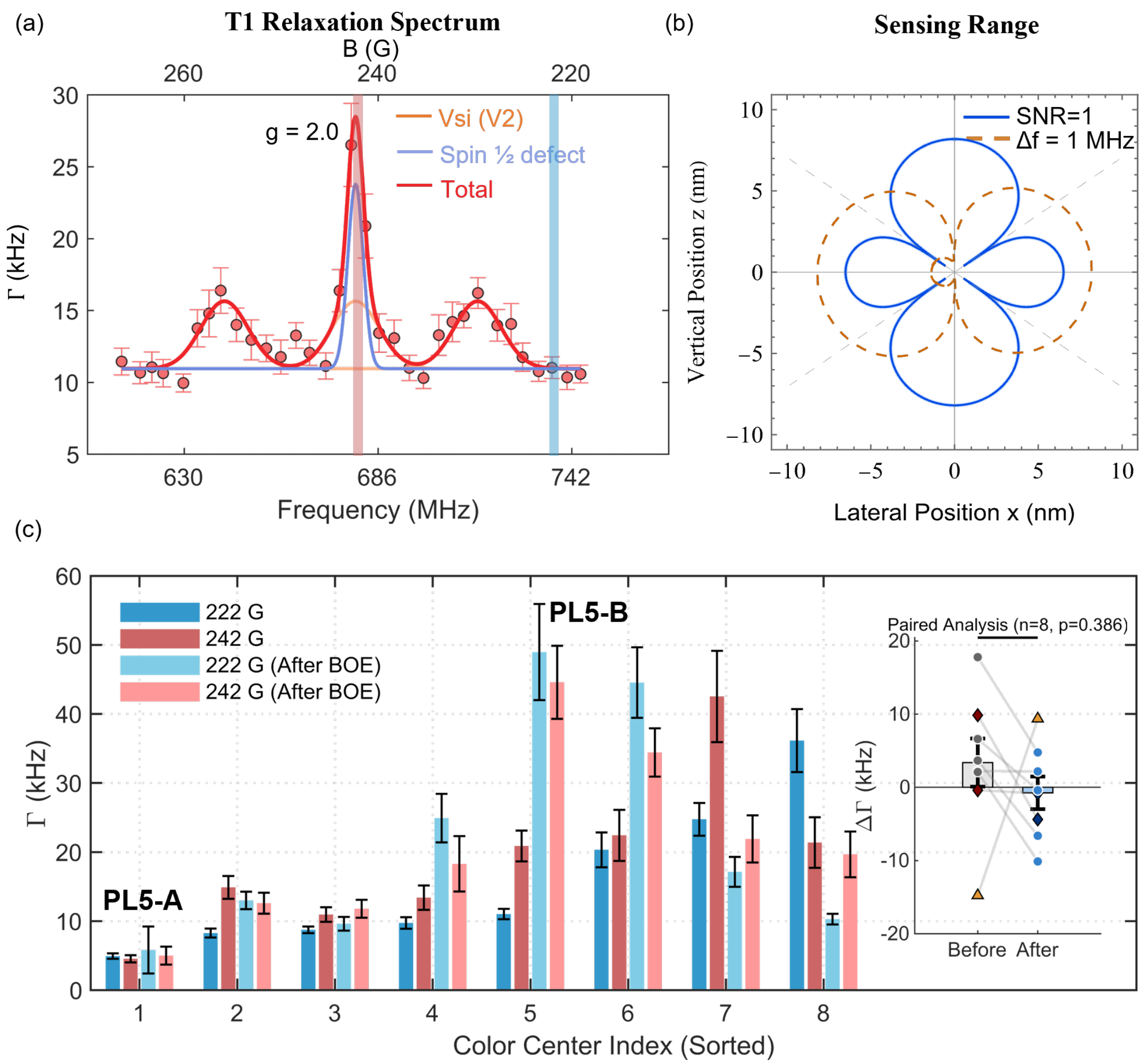

FIG. S10. (a) EPR Spectrum shown in main text. (b) Sensing range of T_1 method. Here, the sensing range boundary is calculated based on the T_1 relaxation measurement protocol. Assuming a dark electron spin (S=1/2) with an inhomogeneous linewidth (FWHM) of 6 MHz, the calculated minimum detectable coupling strength is 420 kHz. The blue contour encloses the spatial region where the dark spin can be detected (SNR > 1), yielding a maximum sensing distance of 5∼ 8 nm. This magnetic sensing volume shows a strong spatial overlap with the electrical sensing range derived from Stark shifts Figure S5(d), where the dashed line represents the $\Delta f = 1 MHz$ boundary. (c) EPR comparison across different PL5 sensors before and after BOE treatment. Inset figure shows the g = 2.0 signal strength ($\Delta\Gamma = \Gamma_{242} - \Gamma_{222}$) before/after BOE treatment.

S3. ANALYSIS OF NOISE SPECTROSCOPY

S3.1. Dynamical Decoupling and SQ/DQ measurement of PL5 with different ODMR Stability

We replaced the CW full-spectrum scan with a two-point measurement to achieve higher temporal resolution. We monitored the time evolution of the ODMR contrast at the two half-maximum points of a single spectral peak. Each point pair was acquired in 6 s and we retrieved the peak position from its contrast to calculate the corresponding standard deviation σ_f. Typical results are shown in Figure S11(a), where the two sensors named PL5-A and PL5-B are consistent with the two PL5 used in the main text. We performed dynamical decoupling experiments of varying pulse orders on the color centers presented in the main text. The results and comparation are shown in Figure S11(b-e). As shown, for unstable quantum sensors, XY8 and higher-order dynamical-decoupling sequences can prolong the coherence time, yet they cannot elevate it to the level attained by stable ones. For color centers with stable spectral peaks, named PL5-A and PL5-C, we performed and compared their SQ and DQ relaxation rates [Figure S11(f-g)]. The results show that within the measured frequency range, the ratio of relaxation rates for DQ and SQ transitions remain essentially the same ($\gamma/\Omega \approx 2.4, \Omega \equiv \Omega_+ = \Omega_-$). It should be noticed that for PL5-B, $(\gamma/\Omega)_{@1GHz} \approx 0.9$, much lower than PL5-A/C, which may indicate a broad magnetic noise background.

Besides, since our measurements on PL5 centers involve environments with significant low-frequency (near-DC) noise, we provide a detailed analysis here to verify the integrity of the dynamical decoupling (DD) data in the presence of frequency drifts.

Firstly, the XY8 sequence is intrinsically robust against static detuning for arbitrary initial states [45]. In this pulse configuration, pulse imperfections and off-resonance effects only influence the DD fidelity to the second order [46]. Regarding the DD filter function, the resonance peak undergoes only negligible modulation when the detuning is small compared to the Rabi frequency of the control pulses [47]. In our experiments on PL5-B, the Rabi frequency is approximately 6.25 MHz, while the detuning—estimated from the ODMR stability σ_f—is on the order of 0.7 MHz. These parameters fall within the regime discussed in [47], where the spectral weight of the filter function remains largely conserved. This ensures

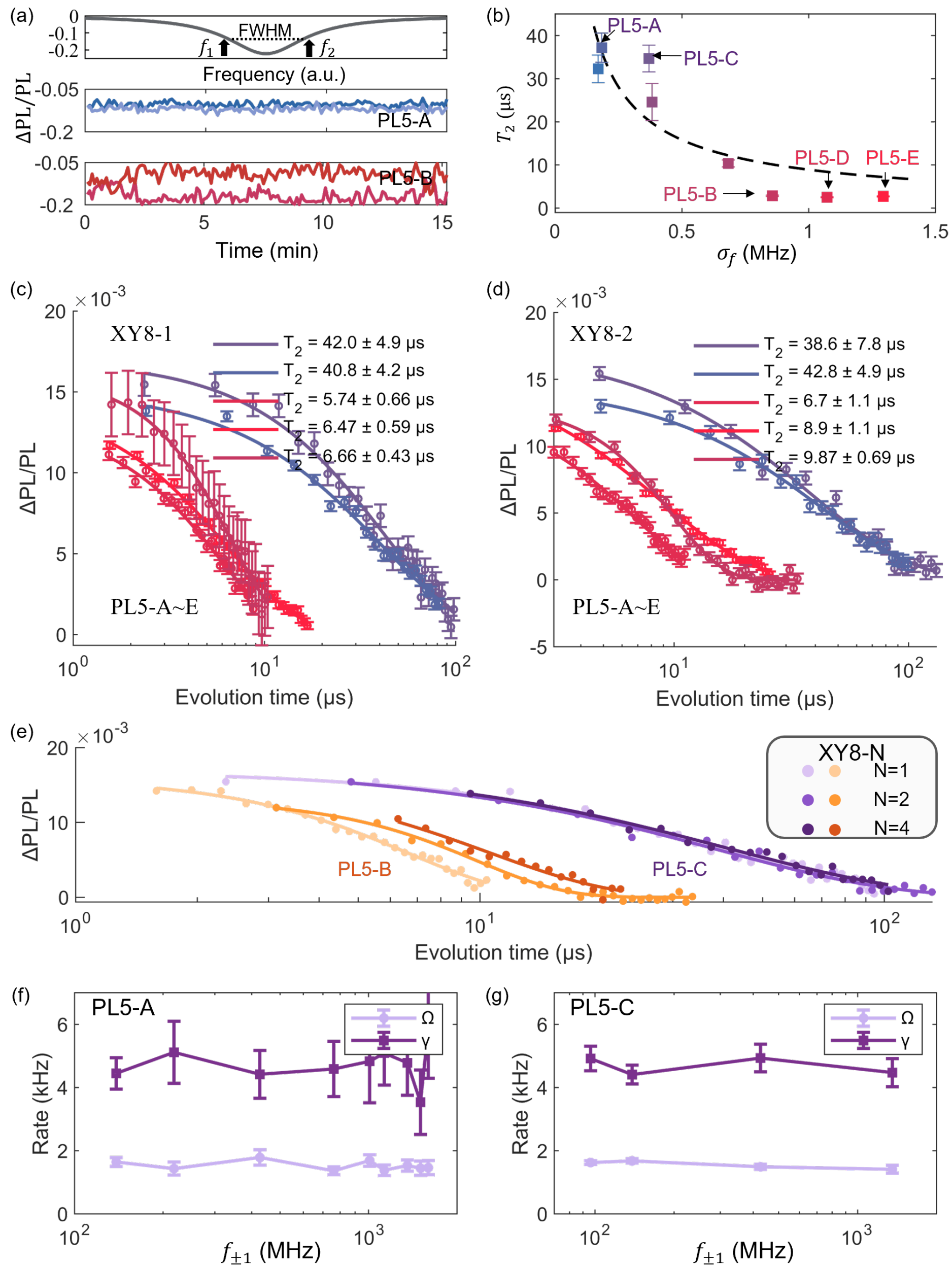

FIG. S11. (a)Two point measurement method and results of PL5-A and PL5-B. The anti-correlation evolution of the contrast at two fixed frequency position f_1 and f_2 reveals different stability behavior of peak position. (b) The relation between coherence time (Hahn echo) T_2 and their ODMR stability σ_f shown in main text. The gray curve serves as a guideline. (c-d) Comparison of dynamical decoupling results at different pulse orders (XY8-1 and XY8-2) between the two types of centers. (e) Coherence enhancement comparision between PL5-B and PL5-C. (f-g) SQ and DQ rate measurement for two stable quantum sensors PL5-A and PL5-C. The relaxation rates show a consistent behavior, remaining essentially the same ($\gamma/\Omega \approx 2.4$).

that the noise spectroscopy $\mathcal{S}(f)$ reconstructed in the main text remains accurate despite slow detuning drifts. Secondly, the frequency drift occurs on a timescale of seconds, which is several orders of magnitude slower than the total duration of a single XY8 sequence ($\sim \mu s$).

Consequently, each individual measurement cycle can be treated under a quasi-static approximation, where the detuning is constant and effectively compensated since XY8 pulse configuration is robust to quasi-static detuning.

S3.2. Comparison of Noise Spectroscopy of PL5-A and B with BOE Treatment

The samples were first cleaned by ultrasonic cleaning procedure in organic solvents and then immersed in a buffered oxide etchant (BOE, 7:1) for 30 s to remove the native oxide layer. Following removal, the samples were rapidly rinsed in deionized water, dried with a nitrogen gun, and quickly immersed in oil to prevent prolonged exposure to the ambient environment. We subsequently relocated the PL5-A and PL5-B sensors to perform comparative measurements. As shown in the Fig. S12, both centers exhibit consistent noise responses following the treatment as desrribed in main text. The coherence time of the PL5-B center under an axial magnetic field was significantly prolonged, enabling the resolution of previously obscured coupled nuclear spin signals that were previously inaccessible due to the limited coherence lifetime. We noted that the influence of the reconstructed noise landscape is slightly less pronounced in PL5-A than in PL5-B, likely due to a difference in their respective depths below the surface.

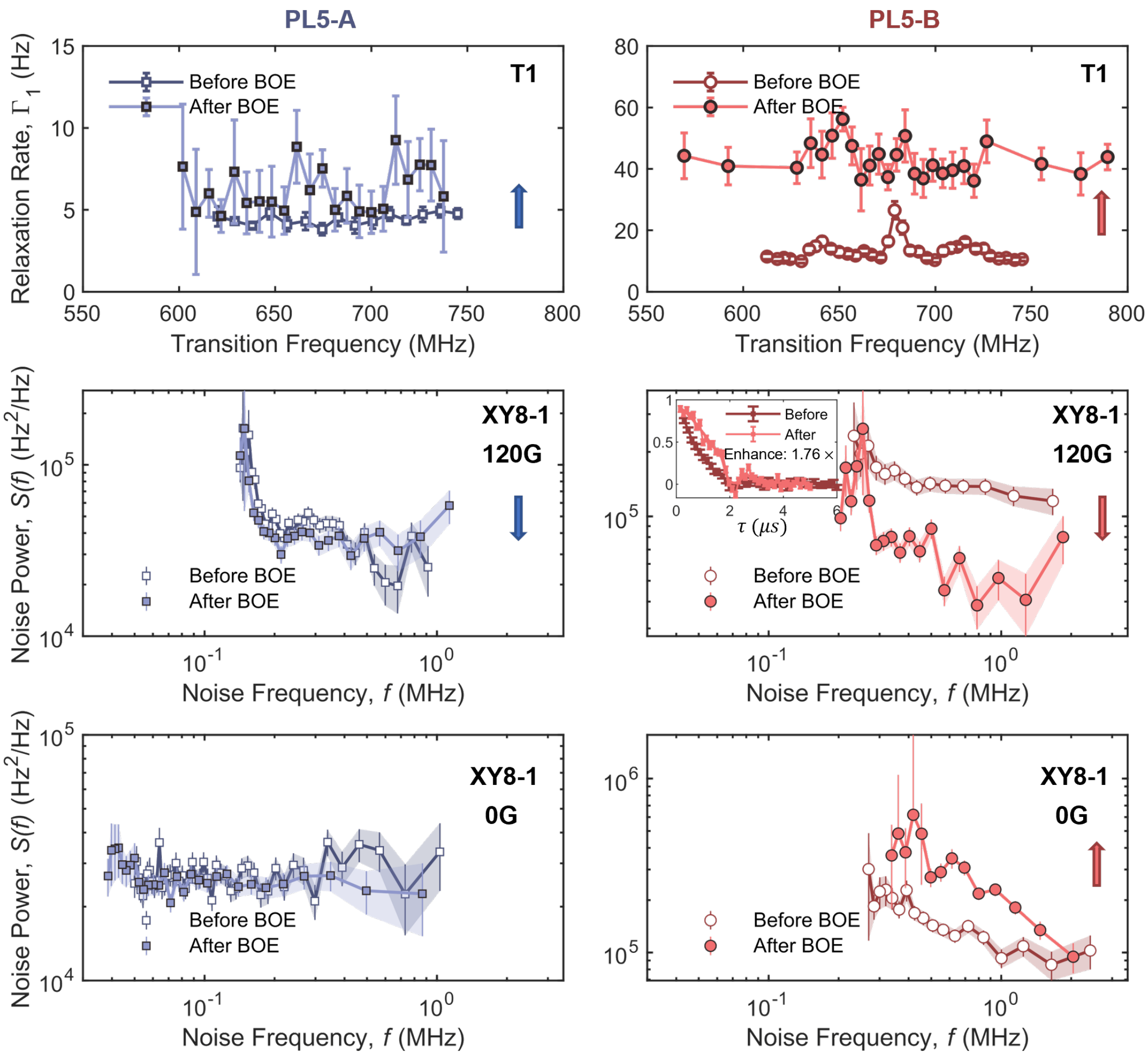

FIG. S12. Frequency-dependent reconfiguration of the noise spectrum following BOE treatment. The removal of the native oxide layer exerts a contrasting impact on the local electromagnetic noise across different frequency bands. T_1 relaxation measurements reveal a significant enhancement of magnetic noise in the GHz regime post-treatment. Conversely, dynamical decoupling under an axial magnetic field demonstrates a pronounced suppression of magnetic noise in the MHz regime. These results highlight the trade-off of surface treatment, where the elimination of the slow-to-intermediate oxide spin bath comes at the cost of increased high-frequency fluctuations from the bare surface.

S3.3. Cross-check of T1 Relaxometry by DEER Measurement

To further examine the local spin-noise fingerprint identified by T_1 relaxometry, we performed a DEER measurement on the same PL5-B center at the magnetic field where the T_1-EPR spectrum shows the V2-related resonance. As shown in Fig. S13, no measurable DEER response is observed for this local target under the present pulse conditions, whereas T_1 relaxometry clearly resolves the corresponding resonance fingerprint. This comparison does not imply that DEER is generally unable to detect local spin environments. Rather,

it reflects the complementary detection channels of the two methods: DEER relies on coherent driving of the target transition, while T_1 relaxometry detects resonant incoherent fluctuations through the sensor longitudinal relaxation channel. Therefore, for the specific local environment examined here, T_1 relaxometry provides access to local dark-spin noise that is not readily resolved by coherent double-resonance detection under our experimental conditions.

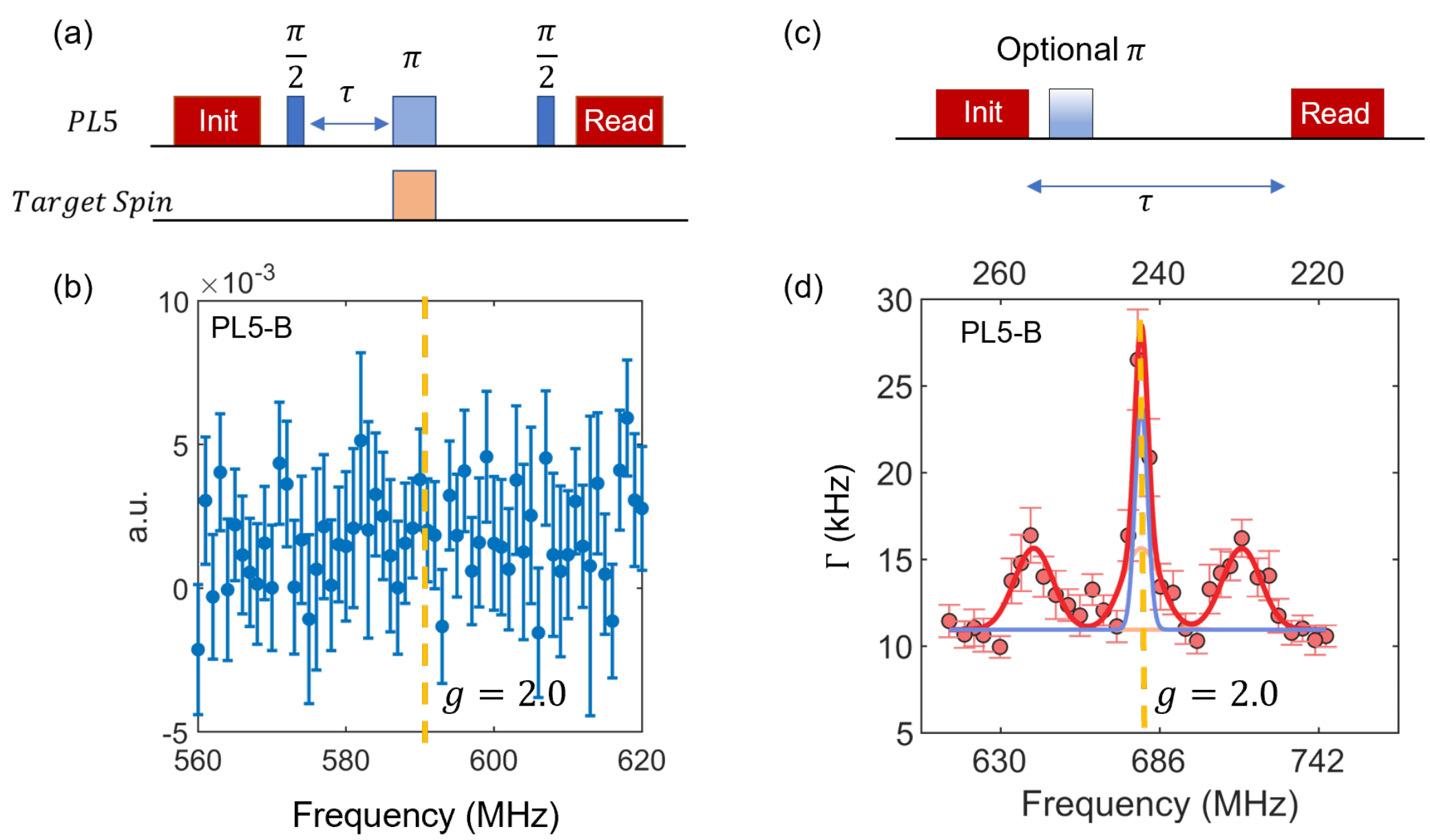

FIG. S13. (a) DEER pulse sequence used to probe the local target spin near PL5-B. (b) DEER spectrum measured on PL5-B. No measurable DEER response is observed under the present pulse conditions. The DEER evolution time is $\tau = 1$ μs. (c) T_1-relaxometry sequence. (d) Corresponding T_1-EPR spectrum of the same PL5-B center, showing a clear resonance fingerprint. The dashed line marks the expected $g = 2.0$ transition.

S4. COMPARISON WITH CONVENTIONAL ENSEMBLE-AVERAGED CHARACTERIZATIONS

To comprehensively evaluate the macroscopic electrical and defect environments of the SiC wafers and to benchmark our PL5 nanoscale sensor against conventional ensemble techniques, standard continuous-wave electron paramagnetic resonance (CW-EPR) measurements, Deep-level transient spectroscopy (DLTS) and electrical property test (I-V and C-V) were performed as discussed below.

S4.1. EPR Measurement

To evaluate the macroscopic defect environments of the SiC wafers, standard continuous-wave electron paramagnetic resonance (CW-EPR) measurements were performed at room temperature. As shown in Figure Fig. S14, both Sample 1 and Sample 2 exhibit a similar volume-averaged resonance centered at $g \approx 2.0027$, a nearly isotropic signal typically attributed to carbon dangling bonds or near-surface amorphous carbon-rich clusters [48, 49]. The undulating baseline observed in the spectra might be attributed to microwave cavity Q-factor degradation and skin depth limitations caused by the highly doped SiC substrates.

Notably, our EPR results do not exhibit distinct characteristic peaks originating from intrinsic vacancy defects. This observation is physically reasonable. In traditional EPR studies, detecting vacancy-related centers typically requires subjecting the samples to high-dose electron irradiation or high-concentration ion implantation to generate defect densities that exceed the macroscopic detection limit [49–52]. Furthermore, specific intrinsic defects, such as the carbon vacancy (V_C), exhibit negative-U properties, whose paramagnetic signals can only be clearly resolved at cryogenic temperatures combined with specific optical excitation [52]. The absence of these signals in our room-temperature measurements highlights the fundamental sensitivity limitations of conventional defect characterization methods.

First, while ensemble ESR provides averaged information over a large number of defects, single PL5 centers reveal pronounced sensor-to-sensor variations within the same wafer, highlighting the heterogeneous nature of the local defect environment.

More crucially, for investigating the specific properties within the epitaxial layer and the interface between epi and SiO_2 layer — which is our primary region of interest, conven-

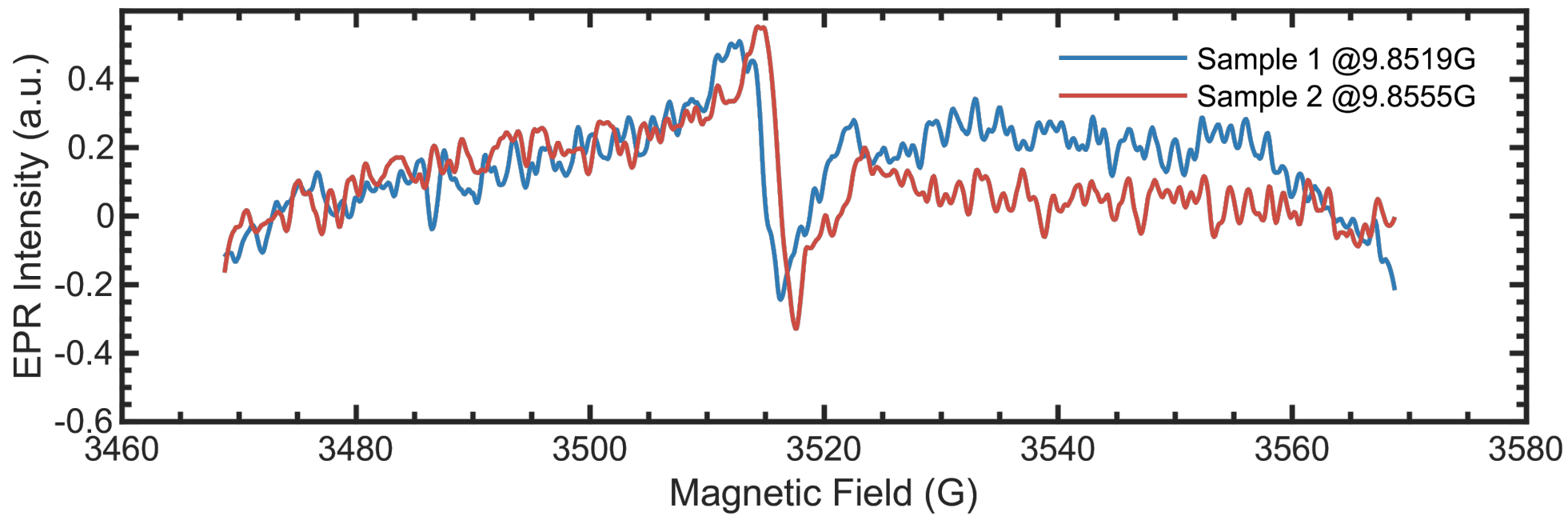

FIG. S14. Room-temperature continuous-wave EPR spectra of the SiC wafers. The EPR spectrum was acquired using a Ciqtek EPR200M X-Band spectrometer. Both Sample 1 and Sample 2 exhibit a similar resonance feature near $g = 2.0027$. The fluctuating baseline is primarily attributed to microwave cavity loading and the skin effect induced by the conductive SiC substrate.

tional EPR is inherently inadequate. Because standard EPR captures a volume-averaged signal from the entire bulk crystal, the weak response of the thin epitaxial layer (tens of micrometers) is completely obscured by the overwhelming background of the thick substrate (hundreds of micrometers)[49]. Consequently, accurately isolating the defect signature of the epilayer using traditional EPR would necessitate the destructive physical removal of the substrate [52]. These inherent bottlenecks of macroscopic characterization demonstrate the unique advantages of nanoscale sensing platform.

S4.2. I-V / C-V characterization

To further characterize the macroscopic electrical properties of the wafers, we utilized two 5×5 mm^2 pieces cut from the exact same Wafer 1 and Wafer 2 used in the main text, without any ion implantation or thermal annealing, named Sample 1-0 and Sample 2-0.

1. Device Fabrication

After identical standard wafer cleaning procedures, a Ni/Au (100 nm / 100 nm) metal stack was deposited on the backside of both wafers, followed by Rapid Thermal Annealing (RTA) at 950°C to form the ohmic contacts simultaneously. Subsequently, a top Ti/Au (10 nm / 150 nm) metal stack was deposited to define the $500\mu m \times 500\mu m$ square Schottky contacts, as shown in Fig. S15(a-b).

2. *Electrical Characterization*

Despite the identical fabrication process, the conventional $I - V$ measurements reveal a stark contrast between the two samples. Sample 2-0 demonstrates near-ideal Schottky diode behavior with a sharp forward turn-on and extremely low reverse leakage current. In contrast, Sample 1-0 exhibits severe non-ideal characteristics, including a significantly delayed turn-on voltage (shifted by nearly 1.0 V), a severely degraded ideality factor, and elevated reverse leakage current. These strongly suggest the presence of a substantial parasitic series resistance or an interfacial barrier layer. The 1 MHz $C - V$ and $1/C^2 - V$ profiles (Fig. S15, Keithley 4200A) provide deeper physical insight into this degradation. For Sample 2-0, the capacitance decreases non-linearly with increasing reverse bias, following the relationship for abrupt junctions. Conversely, the $C - V$ curve of Sample 1-0 exhibits a severe "pinning" effect—the capacitance remains almost entirely flat (∼2.5 pF) regardless of the applied reverse bias up to -20 V. The corresponding $1/C^2$ slope approaches zero, physically implying that the depletion region in the 4H-SiC epitaxial layer fails to expand under an external electric field.

It is worth noting that to ensure the reliability of this difference, we performed mercury-probe capacitance-voltage (MCV) measurements—a conventional technique for wafer inspection—on other bare slices from Wafer 1 and Wafer 2 without metal electrodes Fig. S16. The obtained results were consistent, confirming that the observed surface differences between Wafer 1 and Wafer 2 represent a macroscopic and deterministic variation. This indicates extremely high-density surface states or a thick, non-ideal interfacial layer that completely screens the applied voltage in Wafer 1.

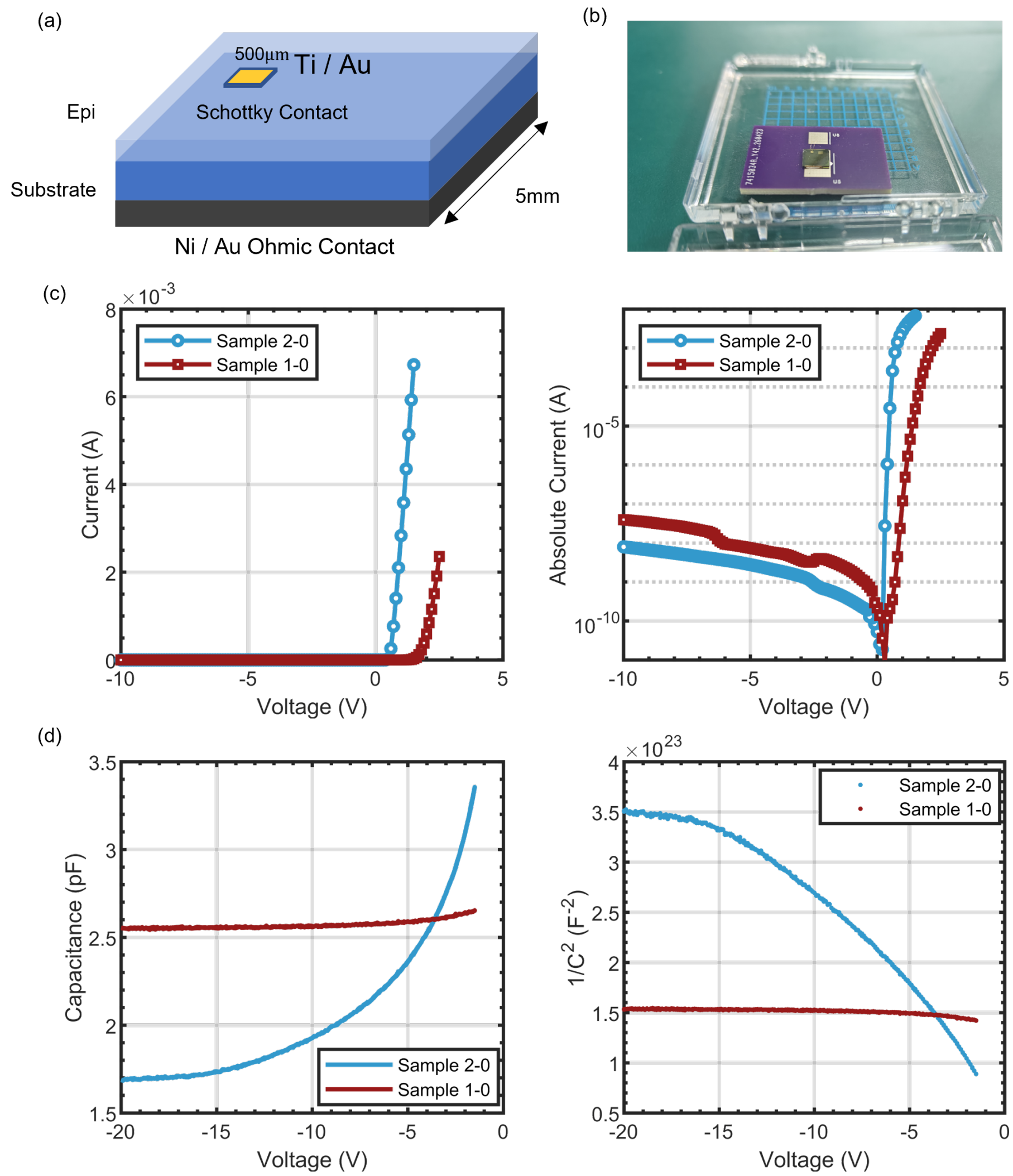

FIG. S15. (a) Schematic of the 4H-SiC Schottky diode structure with top Ti/Au (10 nm / 150 nm, $500 \times 500 \mu m^2$) Schottky contacts and bottom Ni/Au (100 nm / 100 nm) ohmic contacts. (b) Photograph of the fabricated 5×5 mm^2 4H-SiC chips placed in a chip tray. (c) Linear and semi-logarithmic current-voltage ($I - V$) characteristics comparing Sample 1-0 and Sample 2-0 at room temperature. Sample 1-0 exhibits a significantly delayed turn-on voltage, higher ideality factor, and increased reverse leakage current. (d) Capacitance-voltage ($C - V$) and corresponding $1/C^2 - V$ characteristics at 1 MHz under reverse bias. Sample 2-0 shows typical depletion layer expansion, whereas Sample 1-0 exhibits severe capacitance pinning, indicating a high density of interface states or a thick interfacial layer hindering the modulation of the depletion region.

S4.3. DLTS Measurement

Deep-level transient spectroscopy (DLTS) is a powerful technique for characterizing deep-level defects in semiconductor epitaxial layers by monitoring the capacitance transients resulting from the thermal emission of charge carriers following a voltage pulse. Here, we applied DLTS to analyze the defect distribution in our SiC samples. However, for Wafer

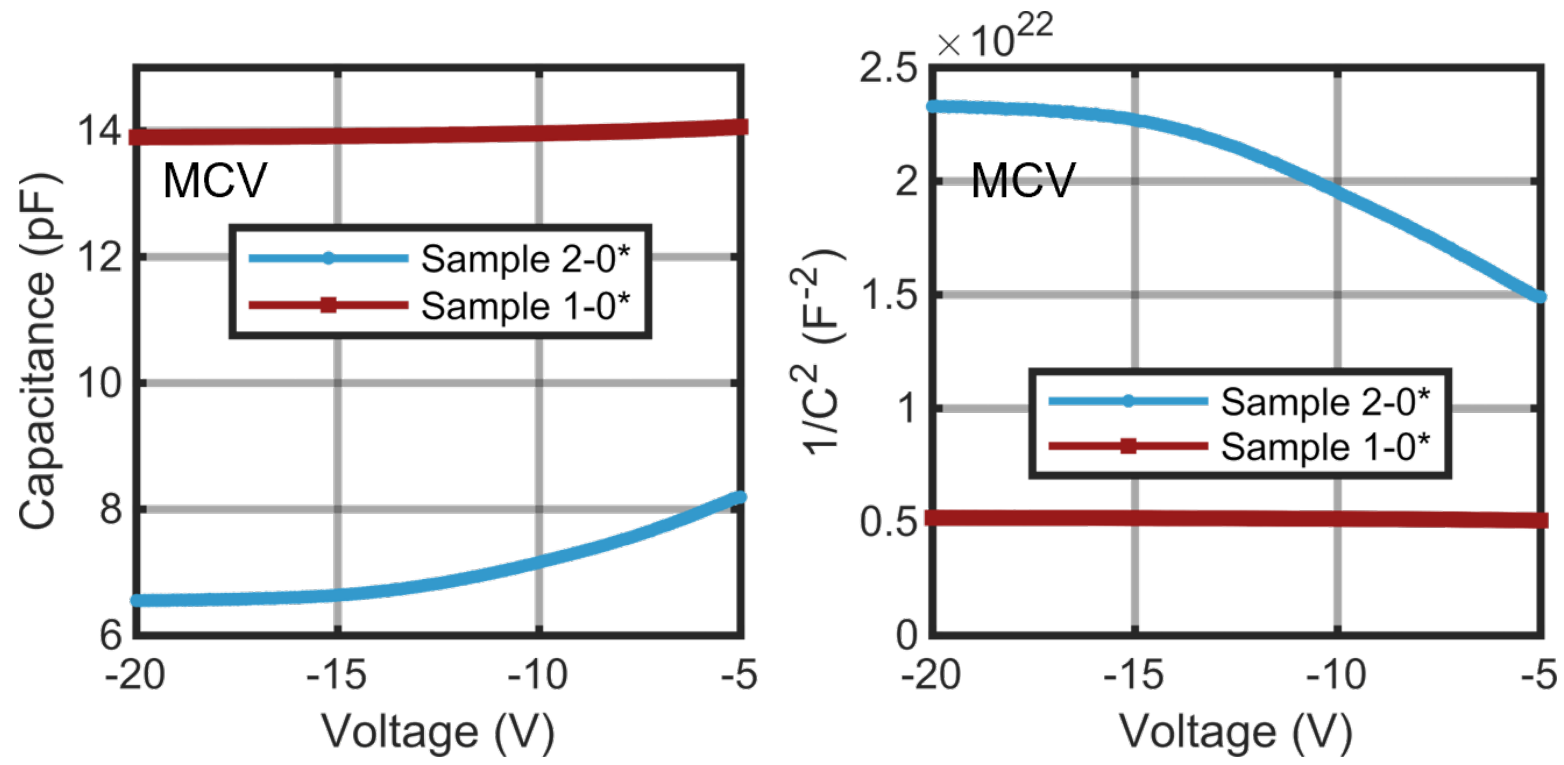

FIG. S16. Mercury-probe capacitance-voltage (MCV) characteristics. $C-V$ and $1/C^2-V$ profiles of bare slices from Wafer 1 (red) and Wafer 2 (blue). Consistent with the fabricated diodes (Fig. S15), the sample from Wafer 1 (corresponding to Sample 1-0*) exhibits severe capacitance pinning, confirming that the severe degradation originates from macroscopic surface deterioration on the wafer itself rather than the fabrication process.

1, the high density of interfacial defects leads to significant pinning of the Fermi level and parasitic capacitance effects, rendering the standard C–V based DLTS measurements totally ineffective. Consequently, we present DLTS results exclusively for Wafer 2 (Fig. S17). Through Arrhenius analysis of the emission rates, we identified characteristic signatures of carbon vacancy ($V_C(Z_{1/2})$) defects within the epitaxial layer [53, 54]. It is important to note that standard C-V-based DLTS depends on a well-defined capacitance response and is therefore less effective for Wafer 1, where the capacitance is strongly pinned. The PL5 quantum-sensing approach, by contrast, directly probes the local interfacial noise environment through an embedded near-surface sensor, revealing nanoscale charge-noise dynamics and nearby spin-related defect fingerprints at room temperature. This provides information complementary to conventional transport-based characterization, particularly in cases where macroscopic capacitance-based methods lose diagnostic sensitivity.

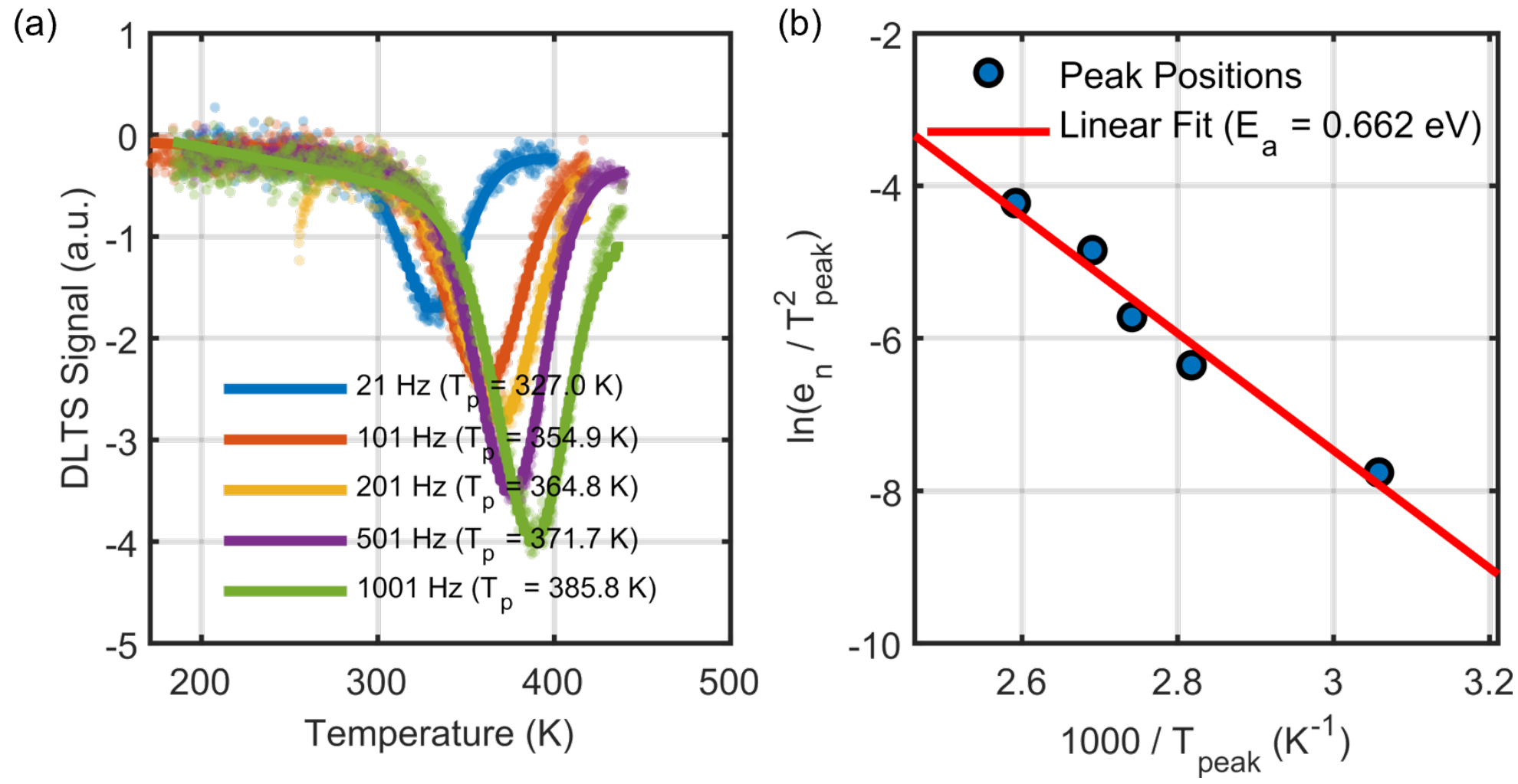

FIG. S17. Characterization of deep-level defects via DLTS on sample 2-0. Deep-level transient spectroscopy (DLTS) scans performed on Wafer 2 using a Semilab DLS-83D spectrometer. Note that measurements on Wafer 1 yielded no signals due to poor interface quality obscuring the capacitance variation. The excitation pulse width was maintained at 20 μs throughout the scans. Arrhenius plots constructed by shifting the lock-in frequencies reveal a prominent trap level whose activation energy closely matches the fingerprints of carbon vacancies ($Z_{1/2}$).

REFERENCES

1. X. She, A. Q. Huang, O. Lucia, B. Ozpineci, Review of silicon carbide power devices and their applications. *IEEE Trans. Ind. Electron.* **64**, 8193–8205 (2017).

2. P.-C. Chen, W.-C. Miao, T. Ahmed, Y.-Y. Pan, C.-L. Lin, S.-C. Chen, H.-C. Kuo, B.-Y. Tsui, D.-H. Lien, Defect inspection techniques in SiC. *Nanoscale Res. Lett.* **17**, 30 (2022).

3. M. E. Bathen, C. T.-K. Lew, J. Woerle, C. Dorfer, U. Grossner, S. Castelletto, B. C. Johnson, Characterization methods for defects and devices in silicon carbide. *J. Appl. Phys.* **131**, 140903 (2022).

4. F. Dolde, M. W. Doherty, J. Michl, I. Jakobi, B. Naydenov, S. Pezzagna, J. Meijer, P. Neumann, F. Jelezko, N. B. Manson, J. Wrachtrup, Nanoscale detection of a single fundamental charge in ambient conditions using the NV-Center in diamond. *Phys. Rev. Lett.* **112**, 097603 (2014).

5. F. Dolde, H. Fedder, M. W. Doherty, T. Nöbauer, F. Rempp, G. Balasubramanian, T. Wolf, F. Reinhard, L. C. L. Hollenberg, F. Jelezko, J. Wrachtrup, Electric-field sensing using single diamond spins. *Nat. Phys.* **7**, 459–463 (2011).

6. H. Jayakumar, J. Henshaw, S. Dhomkar, D. Pagliero, A. Laraoui, N. B. Manson, R. Albu, M. W. Doherty, C. A. Meriles, Optical patterning of trapped charge in nitrogen-doped diamond. *Nat. Commun.* **7**, 12660 (2016).

7. A. Lozovoi, H. Jayakumar, D. Daw, G. Vizkelethy, E. Bielejec, M. W. Doherty, J. Flick, C. A. Meriles, Optical activation and detection of charge transport between individual colour centres in diamond. *Nat. Electron.* **4**, 717–724 (2021).

8. A. A. Wood, D. J. McCloskey, N. Dontschuk, A. Lozovoi, R. M. Goldblatt, T. Delord, D. A. Broadway, J.-P. Tetienne, B. C. Johnson, K. T. Mitchell, C. T.-K. Lew, C. A. Meriles, A. M. Martin, 3D-mapping and manipulation of photocurrent in an optoelectronic diamond device. *Adv. Mater.* **36**, 2405338 (2024).

9. T. Delord, R. Monge, C. A. Meriles, Correlated spectroscopy of electric noise with color center clusters. *Nano Lett.* **24**, 6474–6479 (2024).

10. W. Ji, Z. Liu, Y. Guo, Z. Hu, J. Zhou, S. Dai, Y. Chen, P. Yu, M. Wang, K. Xia, F. Shi, Y. Wang, J. Du, Correlated sensing with a solid-state quantum multisensor system for atomic-scale structural analysis. *Nat. Photonics* **18**, 230–235 (2024).

11. C. F. Weng, S. R. Lin, J. X. Zhao, Y. J. Yang, M. Y. Cai, Y. H. Guo, L. R. Lou, W. Zhu, G. Z. Wang, Tracking single charge jumping with an individual color center in diamond. *Phys. Rev. B* **109**, 224104 (2024).

12. A. Lozovoi, G. Vizkelethy, E. Bielejec, C. A. Meriles, Imaging dark charge emitters in diamond via carrier-to photon conversion. *Sci. Adv.* **8**, eabl9402 (2022).

13. R. Monge, Y. Nakamura, O. Bach, J. Shao, A. Lozovoi, A. A. Wood, K. Sasaki, K. Kobayashi, T. Delord, and C. A. Meriles, Beyond ensemble averaging: Parallelized single-shot readout of hole capture in diamond. arXiv:2507.11722 [cond-mat.mes-hall] (2025).

14. J. Du, F. Shi, X. Kong, F. Jelezko, J. Wrachtrup, Single-molecule scale magnetic resonance spectroscopy using quantum diamond sensors. *Rev. Mod. Phys.* **96**, 025001 (2024).

15. L. Oberg, M. De Vries, L. Hanlon, K. Strazdins, M. S. Barson, M. Doherty, J. Wrachtrup, Solution to electric field screening in diamond quantum electrometers. *Phys. Rev. Appl.* **14**, 014085 (2020).

16. C. L. Degen, F. Reinhard, P. Cappellaro, Quantum sensing. *Rev. Mod. Phys.* **89**, 035002 (2017).

17. C. P. Anderson, A. Bourassa, K. C. Miao, G. Wolfowicz, P. J. Mintun, A. L. Crook, H. Abe, J. U. Hassan, N. T. Son, T. Ohshima, D. D. Awschalom, Electrical and optical control of single spins integrated in scalable semiconductor devices. *Science* **366**, 1225–1230 (2019).

18. Z. Li, X. Guo, Y. Jin, F. Andreoli, A. Bilgin, D. D. Awschalom, N. Delegan, F. J. Heremans, D. Chang, G. Galli, A. A. High, Atomic optical antennas in solids. *Nat. Photonics* **18**, 1113–1120 (2024).

19. G. Pieplow, C. G. Torun, C. Gurr, J. H. D. Munns, F. M. Herrmann, A. Thies, T. Pregnolato, T. Schröder, Quantum electrometer for time-resolved material science at the atomic lattice scale. *Nat. Commun.* **16**, 6435 (2025).

20. W. F. Koehl, B. B. Buckley, F. J. Heremans, G. Calusine, D. D. Awschalom, Room temperature coherent control of defect spin qubits in silicon carbide. *Nature* **479**, 84–87 (2011).

21. H. Hu, Y. Zhou, A. Yi, T. Bao, C. Liu, Q. Luo, Y. Zhang, Z. Wang, Q. Li, D. Lu, Z. Liu, S. Xiao, X. Ou, Q. Song, Room-temperature waveguide integrated quantum register in a semiconductor photonic platform. *Nat. Commun.* **15**, 10256 (2024).

22. Q. Li, J.-F. Wang, F.-F. Yan, J.-Y. Zhou, H.-F. Wang, H. Liu, L.-P. Guo, X. Zhou, A. Gali, Z.-H. Liu, Z.-Q. Wang, K. Sun, G.-P. Guo, J.-S. Tang, H. Li, L.-X. You, J.-S. Xu, C.-F. Li, G.-C. Guo, Room-temperature coherent manipulation of single-spin qubits in silicon carbide with a high readout contrast. *Natl. Sci. Rev.* **9**, nwab122 (2022).

23. Y. Chen, Q. Zhang, M. Liu, J. Wu, J. Liu, X. Zhao, J. Zhou, P. Yu, S. Lin, Y. Teng, W. Yu, Y. Wang, C. Duan, F. Shi, Oxygen-vacancy quantum spin de fects in silicon carbide. arXiv:2504.07558 [cond-mat.mtrl-sci] (2026).

24. A. L. Falk, P. V. Klimov, B. B. Buckley, V. Ivády, I. A. Abrikosov, G. Calusine, W. F. Koehl, Á. Gali, D. D. Awschalom, Electrically and mechanically tunable electron spins in silicon carbide color centers. *Phys. Rev. Lett.* **112**, 187601 (2014).

25. C. Zeledon, B. Pingault, J. C. Marcks, M. Onizhuk, Y. Tsaturyan, Y. xin Wang, B. S. Soloway, H. Abe, M. Ghezellou, J. Ul-Hassan, T. Ohshima, N. T. Son, F. J. Heremans, G. Galli, C. P. Anderson, D. D. Awschalom, Minute-long quantum coherence enabled by electrical depletion of magnetic noise. arXiv:2504.13164 [quant-ph] (2025).

26. T. Steidl, P. Kuna, E. Hesselmeier-Hüttmann, D. Liu, R. Stöhr, W. Knolle, M. Ghezellou, J. Ul-Hassan, M. Schober, M. Bockstedte, G. Bian, A. Gali, V. Vorobyov, J. Wrachtrup, Single V2 defect in 4H silicon carbide Schottky diode at low temperature. *Nat. Commun.* **16**, 4669 (2025).

27. Y.-K. Tzeng, P.-C. Tsai, H.-Y. Liu, O. Y. Chen, H. Hsu, F.-G. Yee, M.-S. Chang, H.-C. Chang, Time-resolved luminescence nanothermometry with nitrogen-vacancy centers in nanodiamonds. *Nano Lett.* **15**, 3945–3952 (2015).

28. R. S. Schoenfeld, W. Harneit, Real time magnetic field sensing and imaging using a single spin in diamond. *Phys. Rev. Lett.* **106**, 030802 (2011).

29. B. A. Myers, A. Ariyaratne, A. C. B. Jayich, Double quantum spin-relaxation limits to coherence of near surface nitrogen-vacancy centers. *Phys. Rev. Lett.* **118**, 197201 (2017).

30. A. Gardill, M. Cambria, S. Kolkowitz, Fast relaxation on qutrit transitions of nitrogen-vacancy centers in nanodiamonds. *Phys. Rev. Appl.* **13**, 034010 (2020).

31. Z. Zhao, X. Ye, S. Xu, P. Yu, Z. Yang, X. Kong, Y. Wang, T. Xie, F. Shi, J. Du, Sub-nanotesla sensitivity at the nanoscale with a single spin. *Natl. Sci. Rev.* **10**, nwad100 (2023).

32. V. Ivády, J. Davidsson, N. T. Son, T. Ohshima, I. A. Abrikosov, A. Gali, Identification of Si-vacancy related room-temperature qubits in 4H silicon carbide. *Phys. Rev. B* **96**, 161114 (2017).

33. Q. Li, J.-F. Wang, F.-F. Yan, Z.-D. Cheng, Z.-H. Liu, K. Zhou, L.-P. Guo, X. Zhou, W.-P. Zhang, X.-X. Wang, W. Huang, J.-S. Xu, C.-F. Li, G.-C. Guo, Nanoscale depth control of implanted shallow silicon vacancies in silicon carbide. *Nanoscale* **11**, 20554–20561 (2019).

34. P. Fiorenza, F. Giannazzo, F. Roccaforte, Characterization of SiO_2/4H-SiC interfaces in 4H-SiC MOSFETs: A review. *Energies* **12**, 2310 (2019).

35. S. Castelletto, C. T.-K. Lew, W.-X. Lin, J.-S. Xu, Quantum systems in silicon carbide for sensing applications. *Rep. Prog. Phys.* **87**, 014501 (2023).

36. H. Roberts, H. Abudayyeh, X. Li, X. Li, Quantum sensing with spin defects beyond diamond. *ACS Nano* **19**, 22528–22575 (2025).

37. C. Wang, L. You, D. Cobden, J. Wang, Towards two dimensional van der Waals ferroelectrics. *Nat. Mater.* **22**, 542–552 (2023).

38. K. Naydenov, N. Donato, F. Udrea, An advanced physical model for the Coulombic scattering mobility in 4H-SiC inversion layers. *J. Appl. Phys.* **127**, 194504 (2020).

39. Q.-C. Hu, J.-Y. Zhou, S. Ren, Z.-X. He, Z.-H. Hao, R.-J. Liang, W.-X. Lin, X. Han, A. Gali, J.-S. Xu, C.-F. Li, G.-C. Guo, High-yield engineering and identification of oxygen-related modified divacancies in 4H-SiC. *Adv. Mater.* **38**, e73419 (2026).

40. J. F. Ziegler, M. Ziegler, J. Biersack, SRIM—The stopping and range of ions in matter (2010). *Nucl. Instrum. Methods Phys. Res., Sect. B* **268**, 1818–1823 (2010).

41. N. Zarrabi, M. G. Düser, R. Reuter, S. D. Dunn, J. Wrachtrup, M. Börsch, "Detecting substeps in the rotary motors of FoF1-ATP synthase by hidden Markov models," in *Biomedical Optics (BiOS) 2007*, J. Enderlein, Z. K. Gryczynski, Eds. SPIE, (2007) pp. 64440E.

42. F. M. Puglisi, P. Pavan, Guidelines for a reliable analysis of random telegraph noise in electronic devices. *IEEE Trans. Instrum. Meas.* **65**, 1435–1442 (2016).

43. S. Lloyd, Least squares quantization in PCM. *IEEE Trans. Inf. Theory* **28**, 129–137 (1982).

44. D. G. Stone, B. Whitefield, M. Kianinia, C. Bradac, Fast characterization of optically detected magnetic resonance spectra via data clustering. *J. Phys. Chem. C* **128**, 13147–13154 (2024).

45. T. Gullion, D. B. Baker, M. S. Conradi, New, compensated Carr-Purcell sequences. *J. Magn. Reson.* **89**, 479–484 (1990).

46. Z.-H. Wang, G. de Lange, D. Ristè, R. Hanson, V. V. Dobrovitski, Comparison of dynamical decoupling protocols for a nitrogen-vacancy center in diamond. *Phys. Rev. B* **85**, 155204 (2012).

47. J. E. Lang, T. Madhavan, J.-P. Tetienne, D. A. Broadway, L. T. Hall, T. Teraji, T. S. Monteiro, A. Stacey, L. C. L. Hollenberg, Nonvanishing effect of detuning errors in dynamical-decoupling-based quantum sensing experiments. *Phys. Rev. A* **99**, 012110 (2019).

48. P. J. Macfarlane, M. E. Zvanut, Characterization of paramagnetic defect centers in three polytypes of dry heat treated, oxidized SiC. *J. Appl. Phys.* **88**, 4122–4127 (2000).

49. Z. C. Feng, R. Hull, R. M. Osgood, J. Parisi, H. Warlimont, Eds., *SiC Power Materials: Devices and Applications*, Springer Series in Materials Science, vol. 73 (Springer Berlin Heidelberg, 2004).

50. N. Mizuochi, S. Yamasaki, H. Takizawa, N. Morishita, T. Ohshima, H. Itoh, J. Isoya, Continuous-wave and pulsed EPR study of the negatively charged silicon vacancy with S=3/2 and C_{3v} symmetry in n-type 4H-SiC. *Phys. Rev. B* **66**, 235202 (2002).

51. N. T. Son, P. N. Hai, E. Janzén, Carbon vacancy related defect in 4H and 6H SiC. *Phys. Rev. B* **63**, 201201 (2001).

52. N. T. Son, X. T. Trinh, L. S. Løvlie, B. G. Svensson, K. Kawahara, J. Suda, T. Kimoto, T. Umeda, J. Isoya, T. Makino, T. Ohshima, E. Janzén, Negative-U system of carbon vacancy in 4H-SiC. *Phys. Rev. Lett.* **109**, 187603 (2012).

53. T. Kimoto, J. A. Cooper, "Characterization techniques and defects in silicon carbide," in *Fundamentals of Silicon Carbide Technology* (John Wiley & Sons Ltd., 2014), pp. 125–187.

54. C. Hemmingsson, N. T. Son, O. Kordina, J. P. Bergman, E. Janzén, J. L. Lindström, S. Savage, N. Nordell, Deep level defects in electron-irradiated 4H SiC epitaxial layers. *J. Appl. Phys.* **81**, 6155–6159 (1997).
